# SVDF: enhancing structural variation detect from long-read sequencing via automatic filtering strategies

**DOI:** 10.1093/bib/bbae336

**Published:** 2024-07-09

**Authors:** Heng Hu, Runtian Gao, Wentao Gao, Bo Gao, Zhongjun Jiang, Murong Zhou, Guohua Wang, Tao Jiang

**Affiliations:** College of Life Sciences, Northeast Forestry University, Harbin 150000, China; College of Life Sciences, Northeast Forestry University, Harbin 150000, China; College of Life Sciences, Northeast Forestry University, Harbin 150000, China; Department of Radiology, The Second Affiliated Hospital of Harbin Medical University, Harbin 150000, China; College of Life Sciences, Northeast Forestry University, Harbin 150000, China; College of Life Sciences, Northeast Forestry University, Harbin 150000, China; College of Computer and Control Engineering, Northeast Forestry University, Harbin 150000, China; State Key Laboratory of Tree Genetics and Breeding, Harbin 150000, China; School of Computer Science and Technology, Harbin Institute of Technology, Harbin 150000, China

**Keywords:** structural variation detection, long-read sequencing, false-positives, deep learning

## Abstract

Structural variation (SV) is an important form of genomic variation that influences gene function and expression by altering the structure of the genome. Although long-read data have been proven to better characterize SVs, SVs detected from noisy long-read data still include a considerable portion of false-positive calls. To accurately detect SVs in long-read data, we present SVDF, a method that employs a learning-based noise filtering strategy and an SV signature-adaptive clustering algorithm, for effectively reducing the likelihood of false-positive events. Benchmarking results from multiple orthogonal experiments demonstrate that, across different sequencing platforms and depths, SVDF achieves higher calling accuracy for each sample compared to several existing general SV calling tools. We believe that, with its meticulous and sensitive SV detection capability, SVDF can bring new opportunities and advancements to cutting-edge genomic research.

## Introduction

Structural variation (SV), a fundamental form of genomic variation, refers to deoxyribonucleic acid rearrangement events larger than 50 bp in size within the genome [1], including deletion (DEL), insertion (INS), duplication (DUP), inversion (INV), and translocation (TRA). Increasing evidence suggests a close association between SV and various human diseases, such as cognitive neurological disorders [2, 3], obesity [4], and cancer [5, 6], as well as their impact on phenotypic traits in many other organisms [7, 8], including growth, development, and adaptive characteristics. Therefore, a comprehensive characterization of the various forms of SVs is crucial to fully understand their contributions to genetic diversity, species differences, and certain phenotypes.

With the rapid development of third-generation sequencing technologies such as Pacific Biosciences (PacBio) [9] and Oxford Nanopore Technologies (ONT) [10], the advantage of better genomic repetitive regions in long-read sequencing (LRS) offers opportunities for higher resolution and more comprehensive SV detection [11, 12]. A study [13] by the Human Genome Structural Variation Consortium revealed that 68% of SVs detected using LRS technologies in 32 human samples could not be identified using short-read data, indicating a significant advantage of LRS in the field of SV detection. However, compared with short-read sequencing, LRS has a relatively higher sequencing error rate, making the accurate characterization of true SVs in noisy LRS data a major challenge.

Several widely used SV detection methods [14–19] based on the LRS have been developed to date, with alignment-based methods being the mainstream approach. These methods typically infer SVs from alignment data using manually designed heuristic rules based on the characteristics of the sequencing platform (such as molecular length and sequencing errors) and different types of SV events. However, manually designed heuristic rules are prone to mistakes in sequencing or alignment-generated noise as genuine SV signals. Considering the inherent complexity of SV events, these noises would interfere with the distribution of real SV signatures and thus affect the prediction of breakpoints and lengths of nearby true events during the signatures clustering process. Therefore, relying solely on manually driven SV detection is challenging. In contrast, deep learning methods, which learn complex abstract features from large annotated datasets, can better capture the hidden features and patterns of SVs, thereby improving detection accuracy [20–22]. Some studies have applied deep learning methods to SV detection. These algorithms transform the SV-related alignment information into image modalities or alignment features, thereby constructing deep learning networks to learn accurate representations of SVs. Nevertheless, fully relying on deep learning methods makes it difficult to balance performance and efficiency. For instance, SVision [20] designed for complex SVs and Cue [21] designed for short-read sequencing data require more time-consuming alignment feature extraction and transformation and can only detect INDELs, such as MAMnet [23] and INSnet [24], which also face time consumption issues.

Here, we present SVDF, a method that utilizes learning-based automatically filtering strategy and a redesigned signature-clustering algorithm to accurately and rapidly detect and genotype five types of SVs in LRS data. During SV signature collection and clustering, SVDF applies a deep neural network and random forest model for filtering false-positives and introduces an adaptive clustering algorithm based on SV signatures for SV event prediction. Our experimental results demonstrate that whilst maintaining acceptable computational efficiency, SVDF exhibits outstanding calling performance across various long-read data error rates, particularly in low-coverage data, and the detection of hard-to-call SV types.

## Materials and methods

### The SVDF workflow

**Figure 1:**
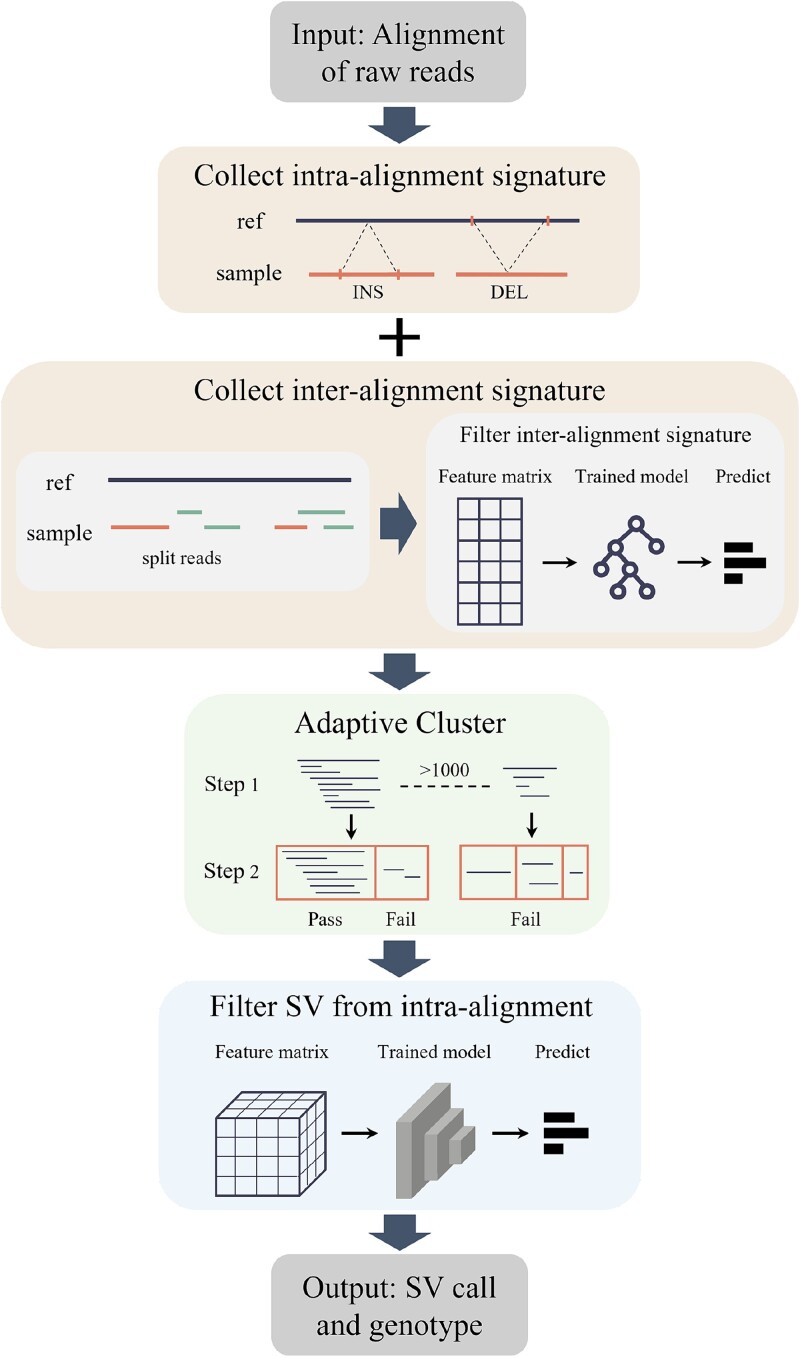
The overall workflow of SVDF. (1) SV signature collection: SVDF comprehensively collects and records various types of SV signatures from intra and inter alignments. (2) Signature clustering: a two-step adaptive clustering algorithm is employed to enhance the signals of true SV events. (3) SV filtering: a CNN is employed to filter false-positive SVs within the clustering results. (4) SV calling and genotype: generates the SV call set and allocates genotypes.

SVDF is a universal SV detection algorithm designed to handle BAM format alignment files generated by various LRS technologies, such as PacBio or ONT. It supports the calling and genotyping of five major SV types: DEL, INS, DUP, INV, and TRA. Figure 1 illustrates the workflow of SVDF. SVDF first extracts SV signatures separately for intra- and inter-alignment regions (Step 1). For intra-alignment signatures, SVDF directly extracts features based on alignment records, whilst for inter-alignment signatures, a trained random forest model is used to filtrate the extracted features to eliminate false positives. All intra and inter signatures are then merged and two-step adaptive clustered approach to identify candidate SV regions by adjusting the stringency of clustering based on local and global contextual information (Step 2). Additionally, SVDF conducts separate filtering operations using convolutional neural networks (CNNs) for fuzzy clustering regions from intra alignment (Step 3). Finally, SVDF genotypes the reported SVs and writes SVs list to an output file in standard VCF format (Step 4).

### Collection of raw SV signatures

The SVDF scans the alignment records of each read to infer signatures from the gap information in the CIGARs (from intra alignment) and split operations during alignment (from inter alignment). During signature inference, the SVDF extracts multiple signature features that are subsequently converted into feature matrices for SV filtering through machine learning.

Specifically, SVDF infers the quadruple information of signatures [SV type, chromosome number, and start and end coordinates, denoted as *(t,c,s,e)*] based on the traditional CIGAR string-matching patterns, which are consistent with previously developed SV callers. For each successfully collected signature, the SVDF recorded 25 filtering features. These features were categorized into three types: global alignment, CIGAR, and base distribution (Supplementary Table S1). Global alignment features include basic information from the alignment records of the read containing the signature, such as alignment quality, soft clipping length, INDELs count, edit distance, etc. Local CIGAR features include inferred information about the start point, length, and type of alignment gaps. Base distribution features include the frequency of each of the four bases in the read containing the signature, and the frequency of repeated bases (defined as a sequence of five consecutive repeats), representing the characteristics of repetitive segments.

For inter-alignment signatures, different algorithms manually design precise heuristic rules and thresholds for various types of SVs to filter out noise data, which is one of the main contributions to the excellent performance of the algorithms. The SVDF applies only basic and lenient heuristic rules to ensure the complete collection of signatures. If the gap between the primary alignment and the supplementary alignment segments is shorter in the read coordinate system than in the reference genome coordinate system, it is inferred as a deletion; otherwise, it is inferred as an insertion. If the two segments are duplicated in the reference genome, it is inferred as a duplication. If the two segments have different alignment orientations or are on different chromosomes, they are inferred as an inversion and a translocation, respectively. During the collection process, SVDF records 22 inter-signature features to train the filtering model. The features were divided into three types: global alignment, split alignment, and base distribution (Supplementary Table S2). Global alignment features and base distribution features record some information from the primary alignment, similar to intra-alignment signatures. Split alignment features record relative information from the primary and supplementary alignments, such as chromosome number consistency, direction consistency, distance in the read, etc.

### Adaptive clustering of raw SV signatures

SV detection typically involves clustering signatures originating from the same SV event to enhance the SV signals. However, because of the ambiguity of clustering thresholds, true events and false-positive SV are often mixed within the same clustering space, affecting the accuracy of SV detection. SVDF employs a two-step adaptive clustering algorithm based on spatial distance and a hierarchical structure. In initial clustering, signatures are rapidly partitioned into different regions according to their coordinates. Subsequently, dynamic hierarchical clustering is performed on the partitioned signatures based on the local and global SV depths of each partition.

For each signature in the form of a quadruple *(t, c, s, e)*, the SVDF first sorts the signatures of the same SV type (*t*) according to the chromosome number (*c*) and start coordinate (*s*). Subsequently, the SVDF scans each signature, and adjacent signatures with a spatial distance greater than 1000 bp (default value) are assigned to different partitions, whereas adjacent signatures with a spatial distance <1000 bp are assigned to the same partition. For two signatures *i* and *j*, the spatial distance for DEL/DUP/INV is defined as *abs(s_j_ – e_i_)*, and the spatial distance for INS/TRA is defined as *abs(s_j_ – s_i_)*. Once rapid partitioning is complete, the SVDF calculates the global SV depth for different SV types based on the number of signatures in each partition:


(1) \begin{equation*} \mathrm{GD}=\frac{1}{N}\sum_{i=1}^N\mathrm{L}{\mathrm{D}}_i \end{equation*}

where LD (local depth) and GD (global depth) represent the SV signal strength within a specific partition and average SV signal strength observed across the entire sample, respectively, and *N* denotes the number of partitions for a specific SV type.

In the second step, SVDF scans each independent partition and calculates the similarity between the signatures within the same partition. Specifically, for two signatures *(t_i_,c_i_,s_i_,e_i_)* and *(t_j_,c_j_,s_j_,e_j_)*, the locus distance *pos_dis* and span distance *span_dis* between signatures *i* and *j* are calculated as follows:


(2) \begin{equation*} \mathrm{pos}\_\mathrm{dis}=\left|\frac{\left({s}_i+{e}_i\right)}{2}-\frac{\left({s}_j+{e}_j\right)}{2}\right| \end{equation*}

(3) \begin{equation*} \mathrm{span}\_\mathrm{dis}=\left|\left({e}_i-{s}_i\right)-\left({e}_j-{s}_j\right)\right| \end{equation*}

Next, the sum of the normalized pos_dis and span_dis according to the two signature spans is transformed into the signature similarity score *S(i, j)*:


(4) \begin{equation*} S\left(i,j\right)=\frac{1}{\max \left({e}_i-{s}_i,{e}_j-{s}_j\right)}\left(\frac{\mathrm{pos}\_\mathrm{dis}}{\lambda }+\mathrm{span}\_\mathrm{dis}\right) \end{equation*}

Here, *λ* (≥1) is a scaling factor that balances the contributions of locus and span distances to the overall similarity score. It is jointly determined by the global depth corresponding to the signature type and the normalized deviation of the local depth of the partition relative to the global depth:


(5) \begin{equation*} \lambda =\frac{\left|\mathrm{GD}-\mathrm{LD}\right|}{\max \left(\mathrm{GD},\mathrm{LD}\right)}+\mathrm{GD} \end{equation*}

Finally, based on a similarity matrix constructed from the similarity scores amongst all signatures within a partition, a hierarchical clustering algorithm is employed to refine the SV signals. The clustering threshold was set to 0.3 (default), indicating that signatures with a similarity of less than 0.3 were further merged into the same cluster. After the subsequent filtering stage is completed, the median start and end coordinates of all the signatures in the remaining clusters are output as the breakpoint coordinates of the candidate SVs.

By adapting the clustering behaviour to the specific characteristics of different genomic regions from different SV and sequencing data types, SVDF optimizes the balance between sensitivity and specificity in SV detection. Amongst them, the *λ* value is crucial in the algorithm, it not only enhances the relative importance of span distance in the measurement of signature similarity (which is particularly important in the scattered distribution of SV signals in CLR and ONT data), but the *λ* value can be dynamically adjusted based on the local and global contextual information to tune the tightness of clustering and alleviate the sensitivity of hierarchical clustering to noise and outliers.

### CNN-based and random forest-based SV filtering models and training

Ignoring the objective errors generated during the sequencing and alignment processes, the noisy data in the alignment-based SV detection process mainly originates from the two steps of signature collection and clustering. SVDF distinguishes the signature features of intra alignment and inter alignment in different steps according to the differences in the sources and characteristics of the noise and builds and trains different network models separately.

The collection of intra-alignment signatures relies on simple string-matching rules. As little noise is generated at this stage, introducing filtering operations hardly achieves the desired effect. In contrast, the ambiguity of the signature similarity distance measurement and the existence of alleles cause signature clustering to produce many false-positive candidate SVs. Simultaneously, the data noise after clustering was amplified, which was more conducive to the model learning of false-positive SV features. Therefore, we selected to filter the noise information of the intra-alignment signature clusters after clustering. Specifically, we first constructed a feature matrix for the signature clusters based on the 25 feature values collected before. For matrices with less than 100 cluster elements, we performed zero-filling operations to ensure that each signature cluster was converted into a feature matrix of fixed size (100, 25). For low-depth signature clusters with depths in the range [1, 20], we treat each signature as a starting point and randomly select a window size smaller than the current cluster depth for data truncation in the row direction to achieve data augmentation. Subsequently, each feature value is scaled to the range of [0, 1] through min-max normalization, and these normalized feature matrices are input into a CNN in batches to learn the differences between true and false-positive samples in local features through convolutional layers of different sizes.

The CNN backbone consisted of three convolutional layers (kernel sizes of 3 × 25, 3 × 8, and 3 × 1), three max-pooling layers, and a fully connected layer (Supplementary Fig. S7). The last three layers are fully connected, and after passing through the normalization and dropout layers, the classification results (DEL/INS/false case) are output. The training uses cross-entropy as the loss function and Adam as the optimisation algorithm, and it adopts a fixed-step learning-rate decay strategy. The batch size for training was set to 256, and the training was stopped after 15 epochs.

The collection of the inter-alignment signatures originates from the gap information produced by the split operation. However, multiple complementary alignment fragments that may be generated by the split cause inconsistencies in the inference of signature information. If these inconsistencies are not filtered, they further increase the subsequent clustering error and affect the calling of true SVs. In addition, the classification boundaries between true and false-positive SV signatures, as well as some different types of true SV signature (such as INS and DUP), are fuzzy, and relying solely on manually designed rules makes it difficult to differentiate between them. Therefore, we employed machine-learning models to classify and filter false-positives from the collected inter-alignment signatures before the clustering step.

We constructed a 22-column raw feature matrix related to inter-alignment signatures, where each row corresponds to a different signature and each column represents a different feature. After min-max normalization, the feature matrix was input into the random forest network for training, and the classification results (DEL/INS/DUP/INV/TRA/false case) were outputted based on the maximum prediction probability. For hyperparameter optimisation, we change the maximum depth of each tree (between 2 and 10), the total number of trees in the forest (between 10 and 100), and the minimum number of leaves required to split an internal node (between 10 and 100) to select the optimal model with the highest F1 score.

### Genotyping

Finally, based on the results of the filtered SV calls, SVDF introduces an optional genotyping module. This module counts the sequencing coverage upstream and downstream of the SV and assigns genotypes based on maximum likelihood probabilities. The maximum likelihood probability model was derived from SVjedi [25], where the data erroneously index of the model was recalibrated based on observations from HG002 and simulated samples. In the CLR and ONT data, the erroneously index is 0.2 for insertion, and is 0.1 for other. For the CCS data, the value is 0.1 for insertion and 0.05 for other.

Details on the generation of the training data and experimental implementation are provided in the supplementary notes of additional file.

## Results

### Benchmarking on simulated sample

**Figure 2:**
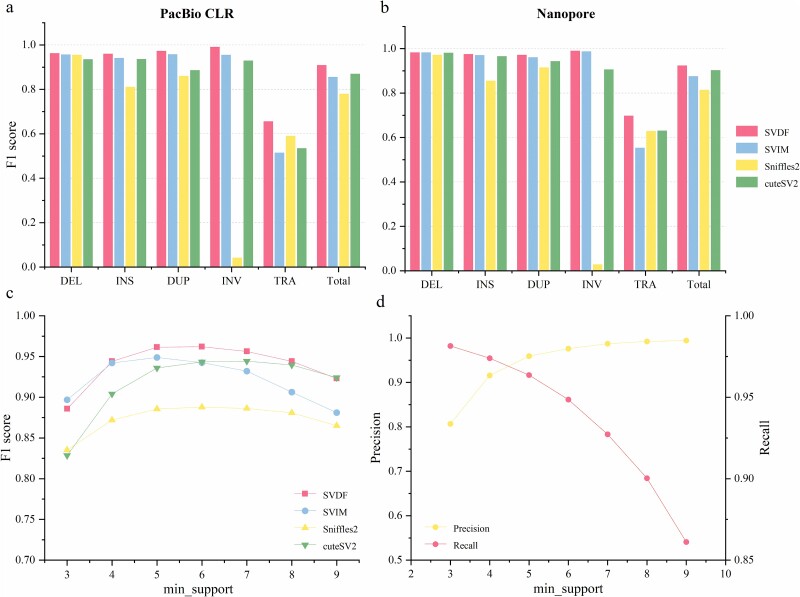
SV detection in simulated sample. (a) and (b) The F1 scores for calling SVs of various SV types by all tools, with CLR data depicted on the left and ONT data on the right. (c) The comparative performance of all tools under different minimum supporting reads thresholds. (d) The trend of precision and recall SV calling by SVDF under different minimum supporting read thresholds.

To comprehensively evaluate the SVDF performance, we first conducted tests on samples simulated using SURVIVOR [26] and PBSIM2 [27] (the implementation of simulate see Methods section). The alignment files of the simulated samples were inputted into the three latest SV calling tools, SVIM, Sniffles2 [28], and cuteSV2 [29], for SV detection. The evaluation results from SUVIVOR demonstrate that amongst the five SV types in PacBio and Nanopore simulated data, SVDF achieved the highest F1 scores compared to the other three calling tools (Fig. 2a). Overall, SVDF obtained 90.9% and 92.4% F1 scores on the PacBio and Nanopore data, respectively, and the second-highest performer was cuteSV2, with 87.0% for PacBio and 90.3% for Nanopore. Sniffles2 showed lower overall F1 scores because of its poor performance in detecting INV. Additionally, SVDF exhibited only a 1.5% difference in SV calling F1 scores between the two LRS data types, which were the least affected by different data platforms compared to the other tools. This suggests that SVDF maintains high calling consistency and stability across different data types (with equal coverage) of the same sample.

Considering the impact of the minimum supporting read value parameter on the results, we manually set a series of ‘min_support’ thresholds and performed repeated calls INDELs on PacBio data to evaluate the SV detection performance under different thresholds. As shown in Figure 2b and c, most SV callers reached their highest F1 scores when the ‘min_support’ was set to 5, indicating the optimal balance between precision and recall. This suggests that approximating the minimum supporting read value based on the read coverage size is reasonable and can effectively filter low-quality SVs. It is worth noting that although some SV callers achieved their best performance at ‘min_support’ thresholds of 6 (Sniffles2) or 7 (cuteSV2), SVDF still outperformed these callers under these parameter conditions. This indicates that the SVDF maintains a high detection performance under different filtering thresholds, demonstrating its robustness when facing different parameter settings.

### Benchmarking on HG002 sample

**Figure 3:**
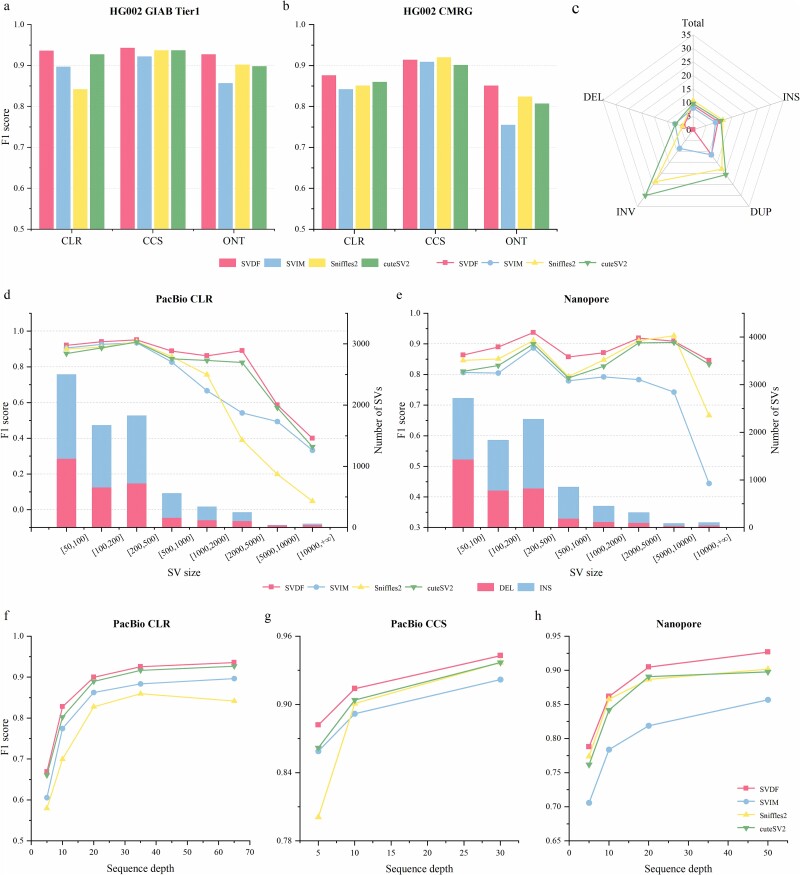
SV detection in the HG002 sample. (a) and (b) Comparison of overall calls F1 scores across Tier1 GIAB genome-wide SV (left) and CMRG benchmark (right). (c) Radar chart of MDR results for the Ashkenazi trio family, including MDR values for overall and different SV types. (d) and (e) The line plot displays the calls F1 scores of various SV sizes in CLR and ONT data by all tools, whilst the bar plot indicates the number of SVs called by SVDF across different SV size ranges. (f–h) Comparison of F1 scores in different down-sampled datasets are illustrated from left to right for CLR, CCS (HIFI), and ONT data.

Next, we evaluated the performance of SVDF on a well-studied HG002 human sample using benchmark sets of deletions and insertions from the high-confidence regions released by the Genome in a Bottle (GIAB) project [30]. The evaluation results from Truvari [31] demonstrated that the SVDF achieved the highest overall F1 scores across all types of test data (Fig. 3a): 93.6% for CLR 65X, 94.3% for CCS 30X, and 92.7% for ONT 50X. The second-best results were 92.7% for CLR in cuteSV2, 93.7% for CCS in Sniffles2 and cuteSV2 and 90.2% for ONT in Sniffles2. The SVDF generally exhibited improvements in deletion detection across all sequencing platforms whilst achieving performance similar to that of cuteSV2 in the more challenging task of calling insertions. We also evaluated the SVDF performance on the GIAB challenging medical gene (CMRG) benchmark set [32], as shown in Figure 3b. In the detection of these complex medically related SVs, SVDF demonstrated a performance comparable to that of Sniffles2 on HIFI data, whilst outperforming other tools in SV calling and genotyping performance on both CLR and ONT data (Supplementary Table S6).

Mendelian inconsistency rate (MDR) is an important metric used in genetic studies to evaluate SV-calling performance. We conducted SV calling on the PacBio CLR sequencing data from all members of the Ashkenazi trio family and calculated the MDR between samples. We found that SVDF’s 8.94% overall MDR is significantly lower than that of the other tools (10.76% for Sniffles2 and 9.63% for cuteSV2), with SVDF having the lowest MDR for all three types of DEL, DUP, and INV (Fig. 3c). A major reason for SVIM’s lower overall MDR (7.97%) was its failure to assign the genotype complex TRA; when excluding TRA from the statistics, MDR of SVDFs decreased to 7.33%, which was lower than that of SVIM. In summary, SVDF demonstrated good MDR performance despite calling for fewer variants (8996, only higher than Sniffles2), primarily because it had the fewest inconsistent SVs (804) between the offspring and parents. This indicates that SVDF effectively filters false-positive SVs.

We further evaluated the ability of the SVDF to call SVs of different SV sizes on LRS data with high error rates (CLR and ONT). As shown in Figure 3d and e, for both the CLR and ONT data, SVDF exhibited the most stable performance in calling SVs, with F1 scores surpassing those of the other tools for almost all SV size ranges. Additionally, based on the distribution of SV sizes, called SVDF, it was observed that SV sizes were mainly concentrated between 50 and 500 bp, and within this range, the performance differences amongst all tools were relatively small. When SV sizes exceeded 500 bp, all tools faced challenges in maintaining high performance owing to the limitations inherent in alignment-based SV calling methods for detecting large SVs (as indicated by the bar chart distribution, primarily originating from insertion), especially on CLR data. However, only SVDF and cuteSV2 maintained relatively high calling F1 scores, indicating that, compared to other alignment-based calling tools, SVDF can detect large insertions more accurately.

To compare the performance of the tools across different coverage datasets, we using Samtools to randomly down sampled the alignment files of the HG002 sample to a series of depths based on the original coverage. Overall, all tools exhibited a decreasing calling performance trend on these down-sampled datasets, but SVDF consistently maintained the highest F1 scores (Fig. 3f–h). Specifically, the SVDF achieved stable F1 scores exceeding 90% on CLR and ONT data with coverage above 20X, as well as CCS data with coverage above 10X. In comparison, achieving this level of performance was challenging for tools other than cuteSV2. We further conducted benchmark tests for genotype accuracy using down-sampled HG002 data. Regardless of the original or lower coverage data, both SVDF and cuteSV2 demonstrated higher genotype F1 scores than SVIM and Sniffles2 (Supplementary Table S5). Specifically, SVDF achieved the highest genotype accuracy on the CCS and ONT datasets, whereas cuteSV2 slightly outperformed SVDF on the CLR dataset. In conclusion, these results indicate that SVDF can accurately identify deletions and insertions, the two major types of SVs, and can reliably call SVs under low-cost sequencing conditions (low-sequencing coverage).

### Validation through assembly based call set on the CHM13 sample

**Figure 4:**
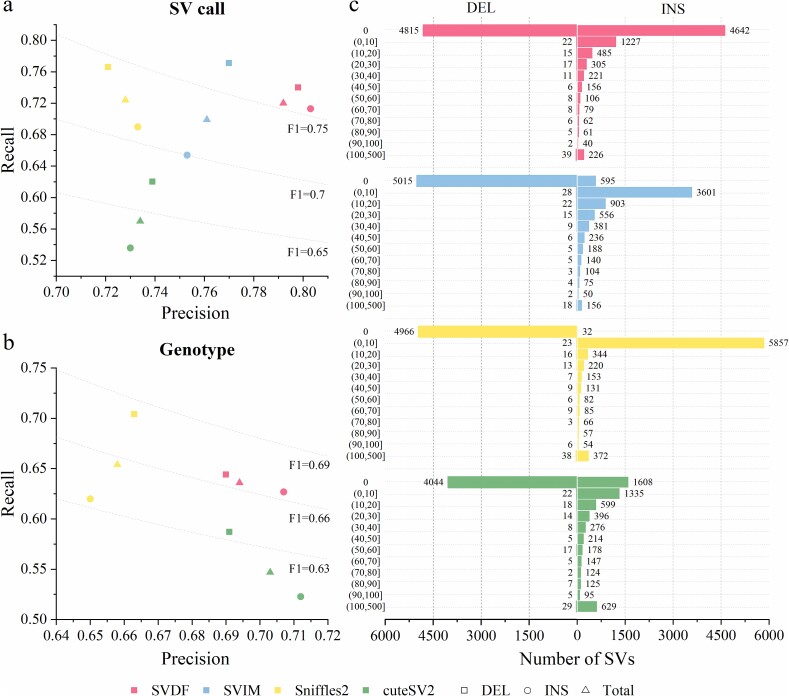
SV detection in the CHM13 sample. (a) and (b) Precision–recall graphs of all tools for SV calling and genotyping in the CHM13 sample compared to the assembly based method (SVIM is hidden in genotyping because of its low performance). (c) The distribution of breakpoint deviation for SVs successfully called by all tools in the CHM13 sample, with the horizontal axis representing the number of SV calls and the vertical axis representing the range of breakpoint deviation.

With the maturation of T2T assembly technology, *de novo* assembly of high-coverage sequencing data enables more precise detection of a larger number of SVs than alignment-based calling methods [33–35]. In light of this finding, we called SVs using all tools on the high-coverage Nanopore 126X alignment data of the CHM13 sample and evaluated the performance of the four alignment-based calling tools against the SV call set constructed using Dipcall [36] based on *de novo* assembly, as the benchmark set. As expected, the number of SVs called by all alignment-based methods was lower than that called by Dipcall (Supplementary Table S8). Specifically, SVDF identified 15 683 SVs, whereas Dipcall identified 17 328 SVs. Although SVDF did not detect the highest number of SV events, its superior performance was evident, with an accuracy of 80.1% (4–7.3% higher than other calling tools, see Fig. 4a), as assessed against the benchmark set. Moreover, a high recall rate of 72.5% (0.1–15.5% higher than other tools) signifies success of the SVDF in identifying the highest number of SV events in the benchmark set. Overall, SVDF achieved an F1 score of 76.1%, surpassing those of SVIM (72.9%), Sniffles2 (72.6%), and cuteSV2 (64.2%), indicating the highest consistency between SVDF and assembly based call sets. Subsequently, in the genotype benchmark testing of CHM13 (a homozygous genome with only GT = 1/1 cases), SVDF achieved the highest F1 score of 66.5% (Fig. 4b), and further demonstrating its ability to accurately discern genotypes.

Accurately inferring SV breakpoints from complex fragments with many inconsistent breakpoint coordinates is challenging for SV calling tools. Alignment-based SV calling tools typically locate SV breakpoints based on the mean or median of multiple candidate SV breakpoints. In contrast, *de novo* assembly generates continuous and complete sequences, offering more precise SV breakpoint localization. Therefore, we evaluated the accuracy of SV breakpoint calling using all tools on the CHM13 sample based on assembly inferred high-precision SV breakpoint positions. Specifically, we adjusted the breakpoint allowance deviation parameter (--*refdist*) in the Truvari tool from the default value of 500–0 bp and calculated the true positives for each tool within the range of [0, 100] in 10-unit intervals of breakpoint allowance deviation. The results demonstrated that SVDF reported the highest number of SVs with completely accurate SV breakpoints (0 bp allowance error), totaling 9457, accounting for 60.3% of the call sets (Fig. 4c). In contrast, SVIM, Sniffles2, and cuteSV2 reported 35.2% (5610), 29% (4998), and 41.9% (5651) of SVs, respectively, with accurate breakpoints. Furthermore, all tools reported the majority of deletions with a zero-breakpoint deviation. SVDF achieved higher breakpoint accuracy primarily in insertions, whereas most insertions reported by other tools exhibited breakpoint deviations, particularly in Sniffles2.

### Benchmarking on cancer sample

**Figure 5:**
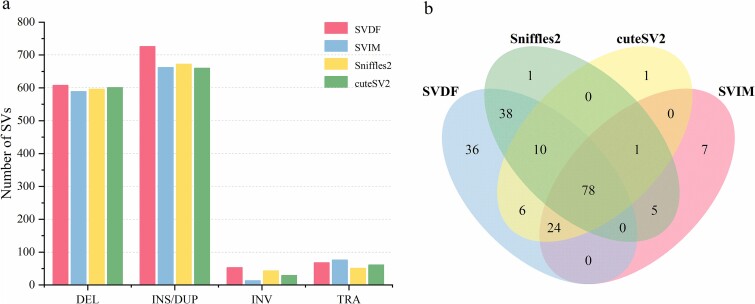
SV detection in the HCC_1395_ cancer sample. (a) The number of successful calls of different types of SV by all tools compared to the HCC_1395_ cancer-specific SV benchmark set. (b) A Venn diagram indicates the overlap of validated SVs called in the HCC_1395_ cancer sample by SVDF and comparative tools, with numbers representing the count of overlaps.

Accurate detection of somatic SV is crucial for cancer target identification, tumour diagnosis, and personalized medicine [5, 37]. However, the number and complexity of SVs in cancer genomes far exceed those in germline genomes [38–40], making them challenging to call. SVDF presents a ‘*sensitive*’ mode designed to call complex SVs in cancer genomes as comprehensively as possible. In ‘*sensitive*’ mode, the SVDF bypasses the filtering modules of general mode, relying solely on custom-designed signature feature matching and a two-step adaptive clustering process to detect SVs. To comprehensively consider calls from multiple sequencing platforms, we evaluated the cancer SV detection capabilities of callers using the union of recall results on the HCC_1395_ cancer-specific SV benchmark set [41] from two call sets generated by each tool. This benchmark set for HCC_1395_ includes 1788 SVs obtained by combining various NGS technologies and multiple SV calling tools to compare tumour and normal cell lines, resulting in a high-confidence consensus. The results showed that SVDF successfully called 608 DELs, 726 INSs/DUPs, 53 INVs, and 68 TRAs from the specificity SV benchmark set, significantly outperforming the other tools in terms of total recall numbers (Fig. 5a). Additionally, 246 specific SVs were validated in HCC_1395_ samples using four methods, including PCR and Affymetrix array technology. Through inspection, it was found that SVDF detected 78.0% (192) of the SVs in the validated SVs list, whereas the detection rates for other tools were 46.7% (115) for SVIM, 54.1% (133) for Sniffles2, and 48.8% (120) for cuteSV2, all notably lower than SVDF, and the Venn diagram (Fig. 5b) also indicates that the SVDF’s call set contains the highest number (36) of unique validated SVs.

Given the limitations of tumour-only calls from tumour-normal paired subtraction analysis for calling cancer-specific SVs, we constructed a novel cancer-specific SV benchmark set based on LRS data from the tumour sample HCC_1395_ and its matched normal cell line, HCC_1395_BL, using the somatic SV calling tool Severus [42] released by the National Cancer Institute (NCI). Severus was specifically designed to call cancer SV by jointly analysing tumour-normal pairs. According to the evaluation results from the Minda tool [42] for somatic SV also developed by the NCI, SVDF called 92.6% of cancer SVs from the Severus benchmark set on PacBio data (Supplementary Table S11), which is 11.3–24.3% higher than similar tools, indicating that SVDF’s cancer SV call set has the highest consistency with the Severus-detected cancer-specific SV call set. On the ONT data, SVDF achieved a similar performance with a recall rate of 83.8%, outperforming the other callers by 4.6–17.2%. Taken together, these results highlight the high sensitivity of SVDF for identifying cancer SVs.

## Discussion

We present a new SV detection tool, SVDF, which improves the SV signal clustering algorithm, automatically filters noise SV events produced at various stages based on a data-driven learning framework, and exhibits a higher accuracy in SV calling performance across different types of LRS platforms, sequencing coverage, and SV types. In addition, SVDF is capable of accurately inferring genotypes and SV breakpoints and supports a more sensitive calling pattern for detecting SVs in cancer genomes. Specifically, the SVDF has three main advantages.

First, the effectiveness of the SVDF adaptive clustering method is one of the primary reasons for its high robustness under various testing conditions. The SVDF automatically adjusts the clustering results based on the characteristics of each SV candidate region without the need to design separate clustering rules or thresholds for different SV types. For regions with a higher local SV depth, SVDF clusters as many high-confidence SV signatures as possible to strengthen the signal, ensuring that potential true SV events are captured rather than being filtered out (Supplementary Figs S1 and S4). Conversely, in regions with lower local SV depths, which contain weaker SV signals or are easily disturbed by potential noise, the SVDF tightens the clustering criteria for better filtering of low-confidence SV signals (Supplementary Fig. S3). Second, SVDF specifically analyses the sources and characteristics of the false-positive distribution in SV detection and trains machine learning models on real and simulated data to filter false positives from intra alignment and inter alignment separately. This learning-based filtering strategy performed well on low-coverage data containing more noise and in the calling of complex SVs (Supplementary Figs S2 and S3). Furthermore, compared to relying entirely on deep learning models, integrating deep learning into the SV detection filtering process and combing the hidden rules learned by neural network models with traditional heuristic rules ensures higher SV calling stability and efficiency. Finally, considering that germline-tailored heuristic rules in traditional methods may not be sensitive enough to capture cancer SVs (Supplementary Fig. S6), we designed a ‘*sensitive*’ mode of SVDF that relies on loose SV signature collection rules and effective signature clustering algorithms to capture cancer SVs more comprehensively and reduce the possibility of missed reports. We believe that the full-spectrum SV call set, accurately detected by SVDF, can be used to supplement areas not covered by short-read sequencing, thereby effectively improving the detection of potential tumour-driving SVs in cancer genomes.

In addition, the choice of SV benchmarks determines the evaluation results of the SV calling tools. The debate over whether the GIAB or the assembly based benchmark set has higher confidence is ongoing [43], but analysing and comparing the results of alignment and assembly based calls offers another perspective for SV evaluation. We found that changes in the evaluation strategy do not significantly affect the SVDF’s excellent call performance. Furthermore, according to the high-precision SV breakpoints detected by the assembly, most SVs called by the SVDF had good breakpoint accuracy owing to their superior clustering and filtering strategies (Supplementary Fig. S5). We also noticed that all calling tools showed a significant decline in performance in assembly based evaluation strategies compared to the GIAB benchmark set, whilst the GIAB evaluation includes known high-confidence regions, another major factor impacting the accuracy of assembly based SV calling results is the presence of reference genome gaps and misassemblies, resulting in the results of SV calling based on the assembly approach not being sufficiently accurate. However, considering the rapid development trend of the assembly approach, it is more appropriate to evaluate alignment-based SV calling tools developed in the future to incorporate assembly-based SV evaluation strategies.

Although a better SV calling performance of the SVDF can be observed in various testing scenarios, it still has some shortcomings that need further improvement. First, although we successfully trained the filtering model on mainstream LRS data, the effectiveness of the filtering still depended on the difference between the real and training samples. In the future, we will introduce more training data and improve the model to cover more populations and variant types. Second, SVDF has taken effective measures to filter out noise signals owing to the limitations of alignment methods, but it still has difficulty detecting some large dispersed insertions. A recent study [44] showed that combining a local assembly with large-insertion detection is an effective method, although it is time-consuming. Finally, because the feature encoding and filtering stages add extra time, the SVDF detection speed was slightly slower than that of Sniffles2 and cuteSV2 (Supplementary Table S12). Fortunately, SVDF employs a multi-thread code construction method, which typically takes only a few minutes to detect SVs in a whole genome using HIFI 30X data of HG002. This speed is significantly faster than traditional deep learning-based SV detection methods, especially in population genomics studies.

**Key Points**

- As a versatile, accurate and fast SV calling tool, SVDF possesses the capability to detect SVs from multiple LRS platforms and various types of SVs with simultaneous assignment of genotypes.
- SVDF integrate machine learning into the SV false-positives filtering process and specially designs an adaptive signatures clustering algorithm, thereby automatically filtering out noise in the traditional SV detection pipeline.
- SVDF can calls complex SVs in cancer genomic data in a ‘sensitive’ mode, providing an effective solution for detecting potential tumour-driven SVs.
- SVDF combines traditional heuristic approaches with deep learning methods, effectively balancing performance and computational resources.

## Supplementary Material

Additional_file_bbae336

## Data Availability

The sequencing data and ground truth call sets in this study are listed in Supplementary Table S13. The SVDF code is available at https://github.com/hh22814/SVDF.
