# Supplementary material for: SVDF: enhancing structural variation detect from long-read sequencing via automatic filtering strategies
Source: Brief Bioinform. 2024 Jul 9;25(4):bbae336. doi: 10.1093/bib/bbae336 (PMC11232458; doi:10.1093/bib/bbae336)
Supplement: Additional_file_bbae336 [file additional_file_bbae336.docx]

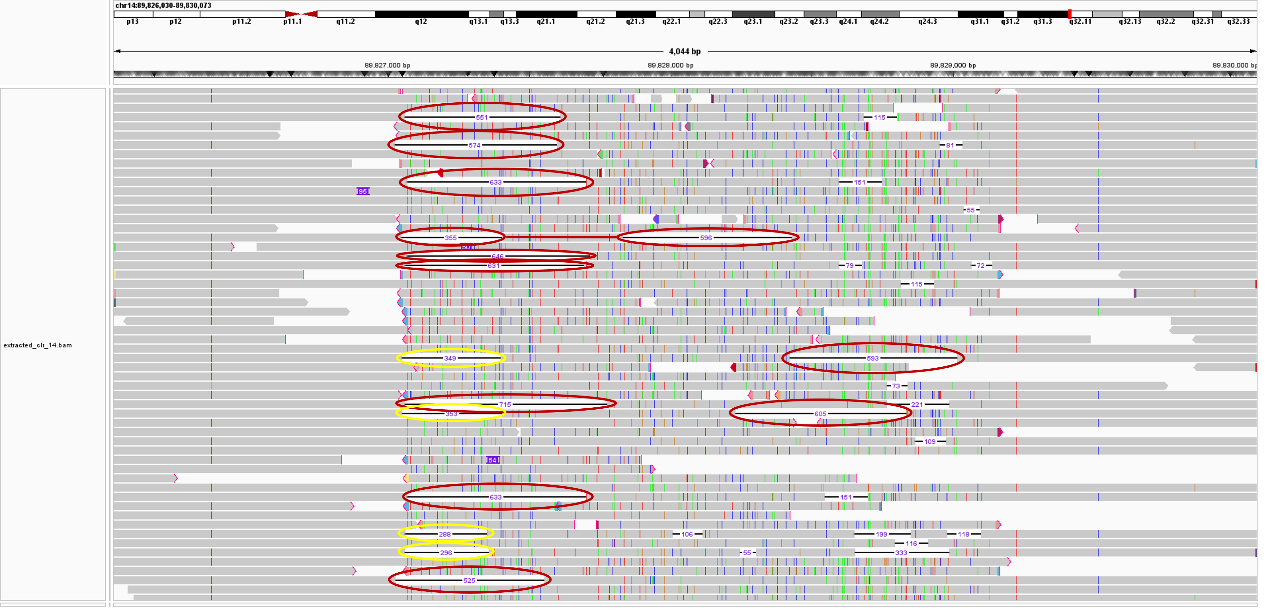


Fig S1. A deletion (Chr14: 89827045, SVLEN=742bp) in the HG002 sample that was successfully called only by SVDF. The IGV screenshot of the CLR 65X data (INDELs smaller than 50bp are hidden) shows that there are multiple deletion signals of different sizes in the region. SVDF clustered these deletion signatures into two candidate SVs of different lengths and filtered out the shorter deletion represented in yellow, which had fewer supporting reads. Finally, SVDF reported an SV of 632 bp within the error margin. In contrast, SVIM called multiple SVs with depths below the minimum supporting reads, resulting in a failed call. Sniffles2 and cuteSV2 reported SVs of 109 bp and 503 bp in length, respectively, exceeding the calling error due to insufficient clustering accuracy.

CLR data

ONT data

CCS data

Fig S2. A false-positive SV (Chr12: 6038800-6039000) in the HG002 sample that was successfully filtered out only by SVDF. The IGV screenshot of the HiFi 10X data shows that there are two reads with a 152 bp insertion signal in the region. However, in reality, there is no evidence supporting the existence of this variant in the benchmark set, CLR, or ONT data. By examining the overall view of the region surrounding this locus, SVDF discovered that the SV signal only originated from two relatively short reads, indicating low reliability, and therefore did not make the call. In contrast, SVIM, Sniffles2, and cuteSV2 all incorrectly called this false-positive insertion. This may be because they did not properly assess the reliability of the evidence supporting the variant and made the SV call based solely on these two reads.


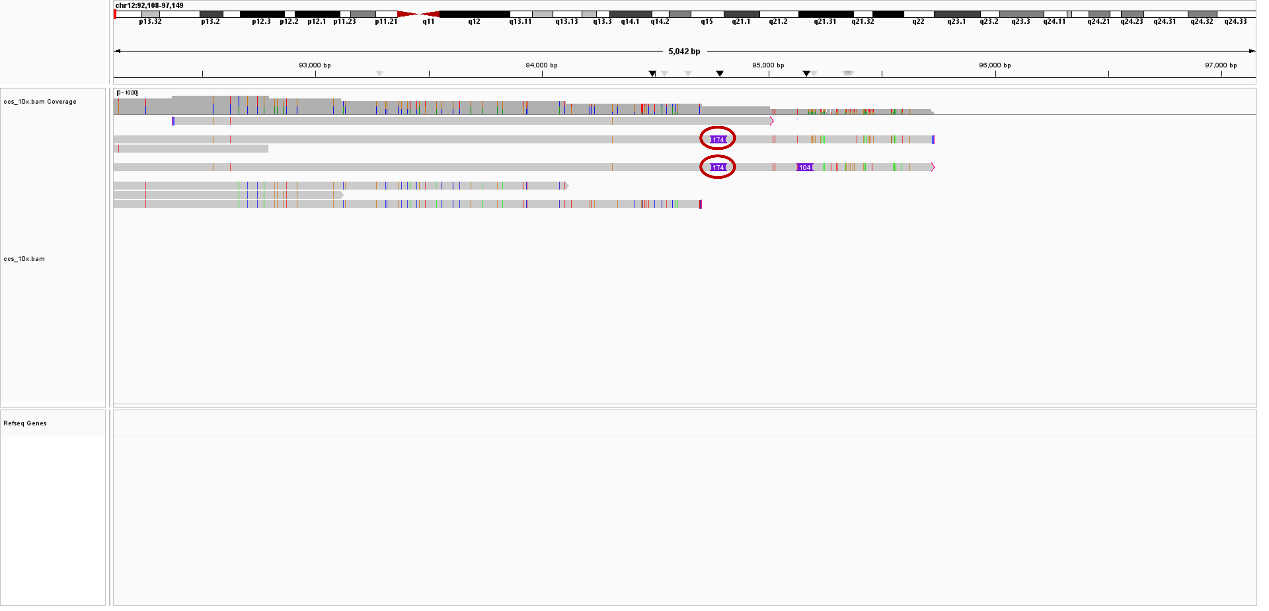


Fig S3. A false-positive SV (Chr12: 94500-95000) in the HG002 sample that was successfully filtered out only by

SVDF. The IGV screenshot of the HiFi 10X data shows that there are two reads with a 174bp insertion signal in the region. However, the features recorded by SVDF indicate that the sequencing depth of this fragment is significantly lower than the overall depth, and the edit distance of the reads (2598) is significantly higher than the normal value. Ultimately, the filtering model successfully filtered out this false positive based on the abnormal alignment features of the region. In comparison, SVIM, Sniffles2, and cuteSV2 all incorrectly called this false-positive insertion. These tools likely relied solely on the presence of the insertion signal in the two reads without considering other important features such as sequencing depth and read alignment quality.

Fig S3. A complex, large insertion (Chr13: 113917459, SVLEN=1178bp) in the HG002 sample that was successfully called only by SVDF. The annotation colors in the IGV screenshot of the ONT 30X data correspond to the clustering categories in the figure below. SVDF merges adjacent signatures on the same read within the region (this strategy has been proven effective in cuteSV) and divides them into three categories through hierarchical clustering. The 1192bp real SV represented in red is retained, while the false-positive SVs represented in yellow and green are filtered out due to not meeting the minimum number of supporting reads. In contrast, SVIM reported two SVs with incorrect lengths near the target SV, while Sniffles2 and cuteSV2 reported one SV each with incorrect lengths of 598bp and 698bp, respectively. This example showcases SVDF's ability to accurately detect complex, large insertions by effectively merging and clustering signatures within the same read.


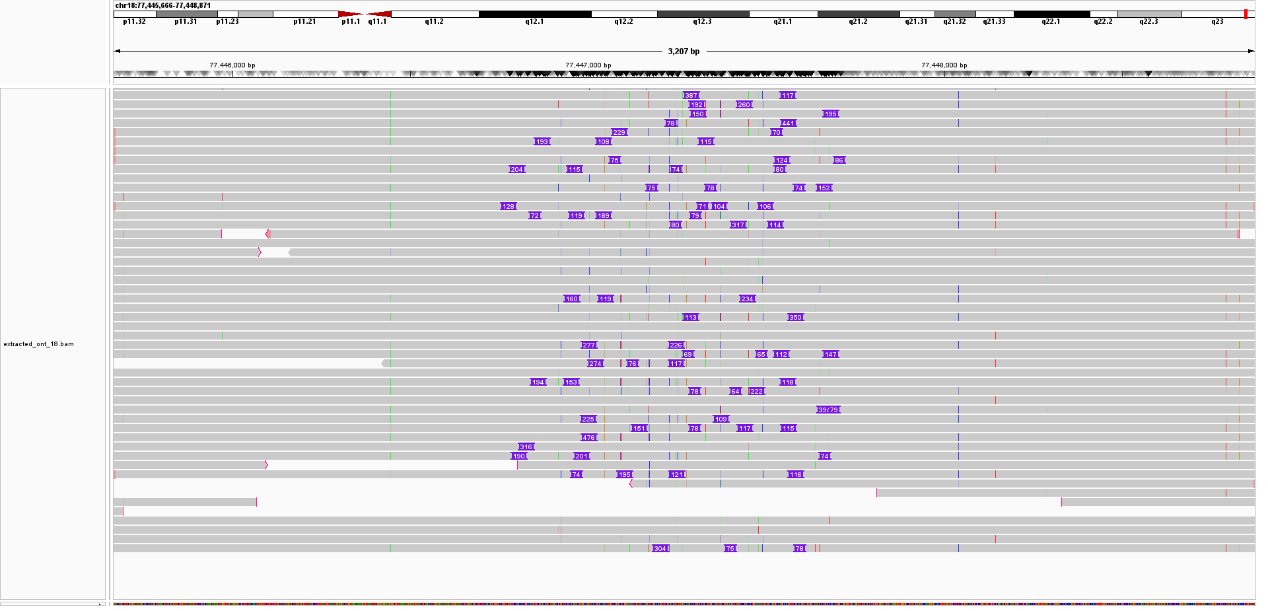


Fig S4. According to the CMRG benchmark set, a complex insertion (Chr18: 77447332, SVLEN=521bp) in the HG002 sample was successfully called only by SVDF. The region contains a large number of scattered small insertions. SVDF restores the original length by merging adjacent signatures and increases the similarity between signatures based on the relatively high local depth in the region, ultimately clustering them into a candidate SV with a length of 459bp. In contrast, SVIM reported two SVs near the region with lengths of 192bp and 114bp, respectively. Sniffles2 and cuteSV2 reported SVs of 195bp and 286bp, respectively, in the vicinity, with lengths significantly lower than the benchmark set results.


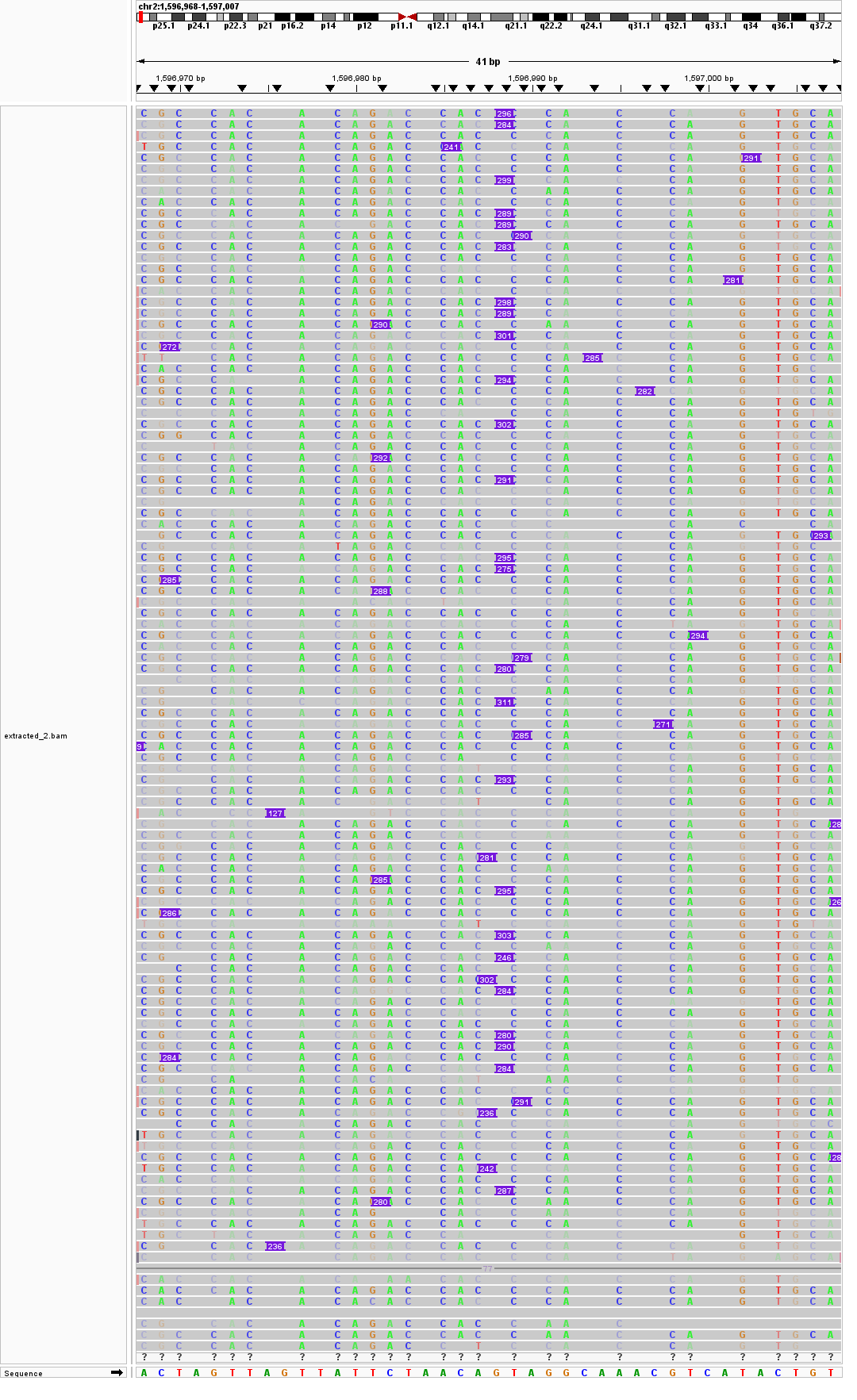


Fig S5. According to the Dipcall benchmark set, an insertion (Chr2: 1596988, SVLEN=292bp) in the CHM13 sample was successfully called with precise breakpoints only by SVDF. SVDF filtered out the noise signals near the region and accurately called the SV at the 1596988bp locus with 0 breakpoint offset. In contrast, the closest SV locus reported by SVIM to the 1596988bp locus was 1597000bp (12bp offset), while Sniffles2 reported 1595941bp (1047bp offset) and cuteSV2 reported 1597053bp (65bp offset).


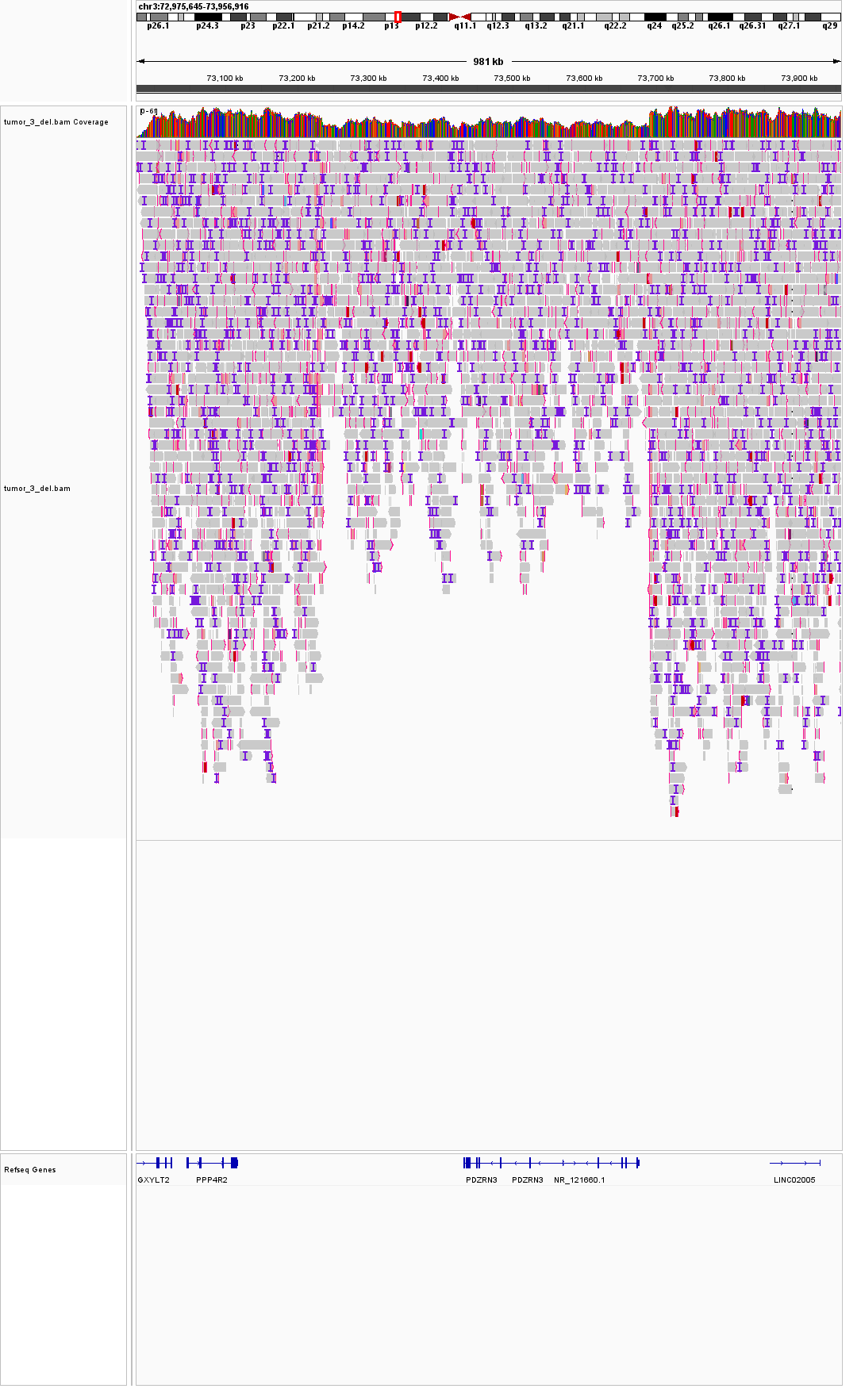


Fig S6. According to the validation list of HCC_1395_ sample, a large cancer-specific deletion (Chr3:73235329, SVLEN=455379bp) that was validated by both BioNano and Affymetrix technology. SVDF reported the SV with a breakpoint error of 0 bp offset. SVIM reported the SV as a wrong translocation type, and Sniffles2 and cuteSV2 calls sets missing this SV record in the neighborhood region.


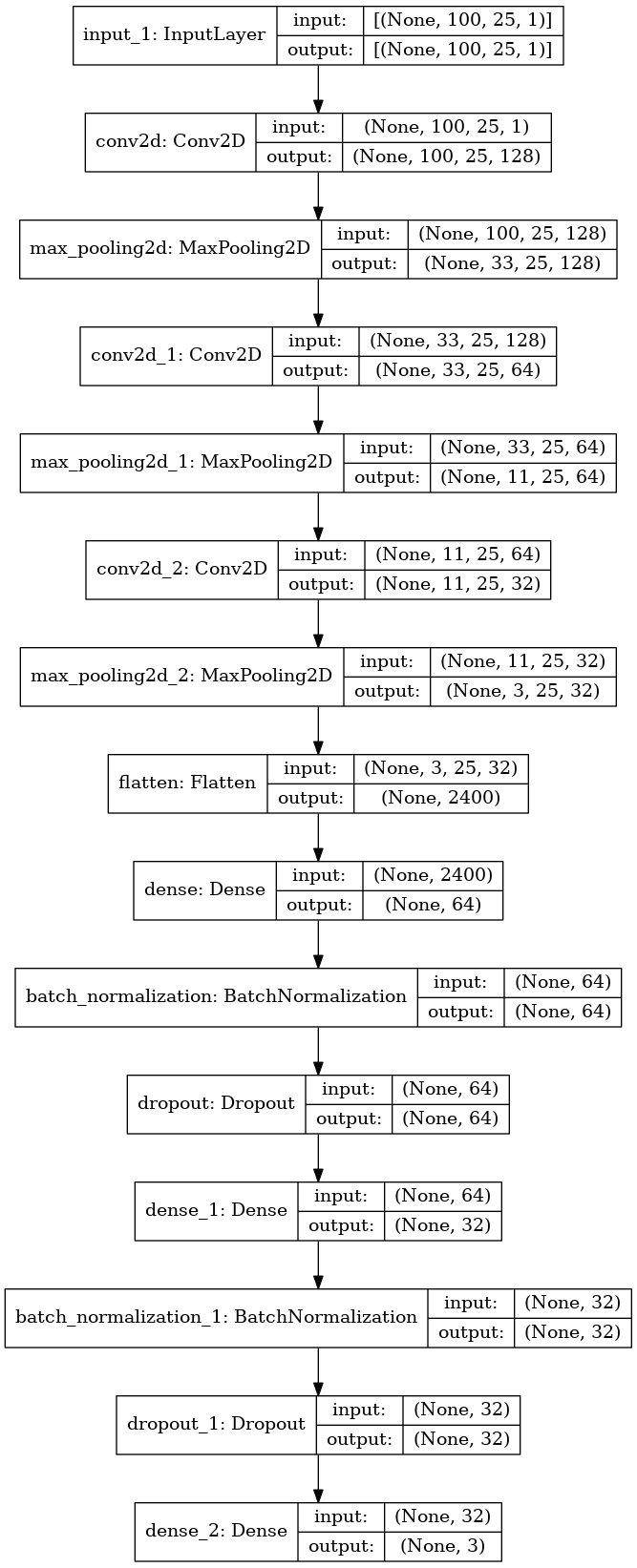


Fig S7. The CNN architecture of the deep learning module for filtering SV from intra-alignment

Table S1 Detailed description of the features of intra-alignment signatures

|  | feature | description |
| --- | --- | --- |
| Global alignment information | MAPQ | Quality score of alignment |
|  | soft_clipped_ratio | Soft clip length accounts for the total length ratio of read |
|  | soft_clipped_length | Total soft clip length of read |
|  | FLAG | FLAG of alignment |
|  | infer_read_length | Infer the length of the read as determined by the alignment tool |
|  | indels_count | The count of INDELs of read |
|  | bin | indexed bins of alignment |
|  | query_alignment_start | start index of the aligned query portion of the sequence |
|  | query_alignment_end | end index of the aligned query portion of the sequence |
|  | reference_id | The chromosome ID in reference genome |
|  | reference_start | The start coordinate in reference genome |
|  | reference_end | The end coordinate in reference genome |
|  | NM_length | The edit-distance between read and reference genome |
|  | total_mismatch | Total length of mismatch between read and reference genome |
| Local CIGARs information | start | The start coordinate of gap in CIGAR strings |
|  | end | The end coordinate of gap in CIGAR strings |
|  | length | The length of gap in CIGAR strings |
|  | pos_ref | The start coordinate of gap in reference genome |
|  | pos_read | The start coordinate of gap in read |
|  | sv_type | Gap type |
| Distribution information of bases | a_freq | The frequency of “A” |
|  | t_freq | The frequency of “T” |
|  | g_freq | The frequency of “G” |
|  | c_freq | The frequency of “C” |
|  | dup_freq | The frequency of AAAAA/TTTTT/GGGGG/CCCCC^^[[1]](#footnote-1)^^ |

Table S2 Detailed description of the features of inter-alignment signatures

|  | feature | description |
| --- | --- | --- |
| Global alignment information | MAPQ | The average alignment quality scores of the primary and supplementary alignments. |
|  | soft_clipped_ratio | Soft clip length accounts for the total length ratio of read |
|  | soft_clipped_length | Total soft clip length of read |
|  | FLAG | FLAG of alignment |
|  | infer_read_length | Infer the length of the read as determined by the alignment tool |
|  | bin | indexed bins of alignment |
|  | query_alignment_start | start index of the aligned query portion of the sequence |
|  | query_alignment_end | end index of the aligned query portion of the sequence |
|  | reference_id | The chromosome ID in reference genome |
|  | reference_start | The start coordinate in reference genome |
|  | reference_end | The end coordinate in reference genome |
|  | NM_length | The edit-distance between read and reference genome |
| Split alignment information | chr_ | Whether the primary alignment and the supplementary alignment are on the same chromosome |
|  | strand_orientation | The consistency of the direction between the primary alignment and the supplementary alignment |
|  | dis_read | The distance between the two alignments in the read coordinate system |
|  | dis_ref | The distance between the two alignments in the reference coordinate system |
|  | deviation | The offset distance between the two alignments in both coordinate systems |
| Distribution information of bases | a_freq | The frequency of “A” |
|  | t_freq | The frequency of “T” |
|  | g_freq | The frequency of “G” |
|  | c_freq | The frequency of “C” |
|  | dup_freq | The frequency of AAAAA/TTTTT/GGGGG/CCCCC |

Table S3 Benchmark results on simulated sample

| Dataset | SV type | SVDF | | | SVIM | | | Sniffles2 | | | cuteSV2 | | |
| --- | --- | --- | --- | --- | --- | --- | --- | --- | --- | --- | --- | --- | --- |
|  |  | P | R | F1 | P | R | F1 | P | R | F1 | P | R | F1 |
| PacBio CLR (30X) | DEL | 0.960 | 0.966 | **0.963** | 0.969 | 0.945 | 0.957 | 0.943 | 0.968 | 0.955 | 0.905 | 0.968 | 0.936 |
|  | INS | 0.959 | 0.962 | **0.960** | 0.958 | 0.926 | 0.941 | 0.695 | 0.973 | 0.811 | 0.911 | 0.963 | 0.936 |
|  | DUP | 1.000 | 0.947 | **0.973** | 1.000 | 0.920 | 0.958 | 1.000 | 0.755 | 0.860 | 0.997 | 0.797 | 0.886 |
|  | INV | 1.000 | 0.983 | **0.991** | 1.000 | 0.915 | 0.955 | 0.529 | 0.022 | 0.042 | 0.884 | 0.980 | 0.929 |
|  | TRA | 0.638 | 0.676 | **0.656** | 0.428 | 0.647 | 0.515 | 0.534 | 0.660 | 0.591 | 0.657 | 0.451 | 0.535 |
|  | Total | 0.906 | 0.913 | **0.909** | 0.833 | 0.881 | 0.856 | 0.757 | 0.816 | 0.78 | 0.882 | 0.860 | 0.870 |
| Oxford Nanopore (30X) | DEL  INS  DUP  INV | 0.985 | 0.980 | **0.983** | 0.984 | 0.982 | **0.983** | 0.972 | 0.972 | 0.972 | 0.980 | 0.984 | 0.982 |
|  |  | 0.970 | 0.980 | **0.975** | 0.970 | 0.971 | 0.971 | 0.760 | 0.978 | 0.856 | 0.951 | 0.981 | 0.966 |
|  |  | 0.989 | 0.956 | **0.972** | 0.984 | 0.939 | 0.961 | 1.000 | 0.844 | 0.915 | 0.995 | 0.896 | 0.943 |
|  |  | 0.993 | 0.988 | **0.990** | 0.990 | 0.985 | 0.988 | 0.333 | 0.015 | 0.028 | 0.835 | 0.990 | 0.906 |
|  | TRA | 0.622 | 0.795 | **0.698** | 0.426 | 0.792 | 0.554 | 0.530 | 0.773 | 0.629 | 0.617 | 0.645 | 0.631 |
|  | Total | 0.903 | 0.946 | **0.924** | 0.820 | 0.941 | 0.876 | 0.784 | 0.84 | 0.814 | 0.892 | 0.915 | 0.903 |

Table S4 Benchmark results of HG002 SV call

| Dataset | Tool |  | DEL |  | INS | | |  | Total |  |
| --- | --- | --- | --- | --- | --- | --- | --- | --- | --- | --- |
|  |  | P | R | F1 | P | R | F1 | P | R | F1 |
| PacBio  CLR 65x | SVDF | 0.978 | 0.956 | **0.967** | 0.945 | 0.885 | **0.914** | 0.959 | 0.914 | **0.936** |
|  | SVIM | 0.970 | 0.935 | 0.952 | 0.949 | 0.778 | 0.855 | 0.959 | 0.842 | 0.897 |
|  | Sniffles2 | 0.970 | 0.948 | 0.959 | 0.693 | 0.870 | 0.771 | 0.790 | 0.902 | 0.842 |
|  | cuteSV2 | 0.978 | 0.920 | 0.948 | 0.943 | 0.885 | 0.913 | 0.957 | 0.899 | 0.927 |
| PacBio  CLR 35x | SVDF | 0.960 | 0.949 | **0.954** | 0.945 | 0.870 | **0.906** | 0.951 | 0.902 | **0.926** |
|  | SVIM | 0.967 | 0.926 | 0.946 | 0.861 | 0.821 | 0.841 | 0.905 | 0.864 | 0.884 |
|  | Sniffles2 | 0.974 | 0.931 | 0.952 | 0.752 | 0.859 | 0.802 | 0.834 | 0.889 | 0.860 |
|  | cuteSV2 | 0.978 | 0.898 | 0.936 | 0.941 | 0.869 | 0.904 | 0.956 | 0.881 | 0.917 |
| PacBio  CLR 20x | SVDF | 0.960 | 0.928 | **0.943** | 0.943 | 0.803 | 0.867 | 0.950 | 0.854 | **0.900** |
|  | SVIM | 0.970 | 0.891 | 0.928 | 0.883 | 0.760 | 0.817 | 0.920 | 0.814 | 0.863 |
|  | Sniffles2 | 0.972 | 0.903 | 0.936 | 0.705 | 0.825 | 0.761 | 0.800 | 0.857 | 0.828 |
|  | cuteSV2 | 0.979 | 0.869 | 0.921 | 0.945 | 0.802 | **0.868** | 0.960 | 0.830 | 0.890 |
| PacBio  CLR 10x | SVDF | 0.949 | 0.888 | **0.917** | 0.931 | 0.638 | 0.757 | 0.940 | 0.740 | **0.828** |
|  | SVIM | 0.977 | 0.757 | 0.853 | 0.884 | 0.603 | 0.717 | 0.925 | 0.666 | 0.775 |
|  | Sniffles2 | 0.965 | 0.832 | 0.894 | 0.504 | 0.735 | 0.598 | 0.638 | 0.775 | 0.700 |
|  | cuteSV2 | 0.978 | 0.741 | 0.844 | 0.942 | 0.657 | **0.774** | 0.958 | 0.692 | 0.803 |
| PacBio  CLR 5x | SVDF | 0.965 | 0.649 | **0.776** | 0.711 | 0.513 | 0.596 | 0.811 | 0.569 | **0.669** |
|  | SVIM | 0.963 | 0.633 | 0.764 | 0.525 | 0.501 | 0.513 | 0.667 | 0.556 | 0.606 |
|  | Sniffles2 | 0.965 | 0.616 | 0.752 | 0.457 | 0.527 | 0.489 | 0.599 | 0.563 | 0.580 |
|  | cuteSV2 | 0.969 | 0.623 | 0.758 | 0.682 | 0.533 | **0.598** | 0.787 | 0.570 | 0.661 |
| PacBio  CCS 30x | SVDF | 0.971 | 0.968 | **0.969** | 0.924 | 0.924 | 0.924 | 0.943 | 0.942 | **0.943** |
|  | SVIM | 0.946 | 0.965 | 0.956 | 0.870 | 0.931 | 0.900 | 0.901 | 0.945 | 0.922 |
|  | Sniffles2 | 0.948 | 0.964 | 0.956 | 0.906 | 0.944 | **0.925** | 0.923 | 0.952 | 0.937 |
|  | cuteSV2 | 0.972 | 0.949 | 0.960 | 0.929 | 0.912 | 0.921 | 0.947 | 0.927 | 0.937 |
| PacBio  CCS 10x | SVDF | 0.968 | 0.918 | **0.942** | 0.924 | 0.864 | **0.893** | 0.943 | 0.886 | **0.914** |
|  | SVIM | 0.938 | 0.918 | 0.928 | 0.859 | 0.877 | 0.868 | 0.890 | 0.894 | 0.892 |
|  | Sniffles2 | 0.946 | 0.891 | 0.918 | 0.903 | 0.875 | 0.889 | 0.920 | 0.882 | 0.901 |
|  | cuteSV2 | 0.972 | 0.881 | 0.924 | 0.933 | 0.851 | 0.890 | 0.949 | 0.863 | 0.904 |
| PacBio  CCS 5x | SVDF | 0.963 | 0.861 | **0.910** | 0.909 | 0.821 | **0.863** | 0.931 | 0.837 | **0.882** |
|  | SVIM | 0.916 | 0.896 | 0.906 | 0.804 | 0.855 | 0.828 | 0.848 | 0.872 | 0.859 |
|  | Sniffles2 | 0.954 | 0.700 | 0.808 | 0.913 | 0.707 | 0.797 | 0.930 | 0.704 | 0.801 |
|  | cuteSV2 | 0.971 | 0.787 | 0.869 | 0.930 | 0.795 | 0.857 | 0.947 | 0.791 | 0.862 |
| Oxford  Nanopore 50x | SVDF | 0.955 | 0.967 | **0.961** | 0.932 | 0.877 | **0.903** | 0.942 | 0.914 | **0.927** |
|  | SVIM | 0.928 | 0.944 | 0.936 | 0.801 | 0.803 | 0.802 | 0.853 | 0.861 | 0.857 |
|  | Sniffles2 | 0.912 | 0.954 | 0.932 | 0.882 | 0.878 | 0.880 | 0.894 | 0.909 | 0.902 |
|  | cuteSV2 | 0.950 | 0.942 | 0.946 | 0.887 | 0.842 | 0.864 | 0.913 | 0.883 | 0.898 |
| Oxford  Nanopore 20x | SVDF | 0.947 | 0.932 | **0.940** | 0.930 | 0.834 | **0.879** | 0.938 | 0.874 | **0.906** |
|  | SVIM | 0.895 | 0.938 | 0.916 | 0.705 | 0.811 | 0.755 | 0.779 | 0.863 | 0.819 |
|  | Sniffles2 | 0.881 | 0.941 | 0.910 | 0.870 | 0.871 | 0.871 | 0.875 | 0.900 | 0.887 |
|  | cuteSV2 | 0.942 | 0.918 | 0.930 | 0.888 | 0.840 | 0.863 | 0.910 | 0.872 | 0.891 |
| Oxford  Nanopore 10x | SVDF | 0.931 | 0.859 | **0.893** | 0.926 | 0.766 | 0.838 | 0.928 | 0.804 | **0.863** |
|  | SVIM | 0.880 | 0.853 | 0.866 | 0.725 | 0.729 | 0.727 | 0.787 | 0.780 | 0.784 |
|  | Sniffles2 | 0.858 | 0.891 | 0.874 | 0.863 | 0.829 | **0.846** | 0.861 | 0.854 | 0.858 |
|  | cuteSV2 | 0.923 | 0.834 | 0.876 | 0.885 | 0.760 | 0.818 | 0.901 | 0.791 | 0.842 |
| Oxford  Nanopore 5x | SVDF | 0.899 | 0.743 | **0.814** | 0.903 | 0.671 | **0.770** | 0.901 | 0.701 | **0.789** |
|  | SVIM | 0.827 | 0.730 | 0.775 | 0.690 | 0.631 | 0.659 | 0.745 | 0.672 | 0.706 |
|  | Sniffles2 | 0.869 | 0.726 | 0.791 | 0.863 | 0.681 | 0.761 | 0.865 | 0.699 | 0.774 |
|  | cuteSV2 | 0.892 | 0.713 | 0.792 | 0.856 | 0.652 | 0.740 | 0.871 | 0.677 | 0.762 |

Table S5 Benchmark results of HG002 SV genotype

| Dataset | Tool |  | DEL |  | INS | | |  | Total |  |
| --- | --- | --- | --- | --- | --- | --- | --- | --- | --- | --- |
|  |  | P | R | F1 | P | R | F1 | P | R | F1 |
| PacBio  CLR 65x | SVDF | 0.949 | 0.928 | **0.938** | 0.923 | 0.842 | 0.881 | 0.934 | 0.877 | 0.905 |
|  | SVIM | 0.926 | 0.892 | 0.909 | 0.722 | 0.590 | 0.649 | 0.814 | 0.714 | 0.761 |
|  | Sniffles2 | 0.929 | 0.908 | 0.918 | 0.649 | 0.814 | 0.722 | 0.747 | 0.853 | 0.796 |
|  | cuteSV2 | 0.960 | 0.903 | 0.931 | 0.920 | 0.864 | **0.891** | 0.937 | 0.880 | **0.907** |
| PacBio  CLR 35x | SVDF | 0.936 | 0.900 | **0.917** | 0.909 | 0.837 | 0.871 | 0.920 | 0.863 | 0.890 |
|  | SVIM | 0.913 | 0.875 | 0.894 | 0.767 | 0.650 | 0.704 | 0.831 | 0.743 | 0.784 |
|  | Sniffles2 | 0.926 | 0.885 | 0.905 | 0.692 | 0.791 | 0.738 | 0.778 | 0.830 | 0.803 |
|  | cuteSV2 | 0.955 | 0.876 | 0.914 | 0.914 | 0.844 | **0.878** | 0.931 | 0.857 | **0.892** |
| PacBio  CLR 20x | SVDF | 0.929 | 0.859 | **0.893** | 0.891 | 0.758 | 0.819 | 0.907 | 0.800 | **0.850** |
|  | SVIM | 0.917 | 0.842 | 0.878 | 0.780 | 0.615 | 0.688 | 0.806 | 0.727 | 0.764 |
|  | Sniffles2 | 0.906 | 0.842 | 0.873 | 0.639 | 0.747 | 0.689 | 0.734 | 0.786 | 0.759 |
|  | cuteSV2 | 0.941 | 0.835 | 0.885 | 0.896 | 0.760 | **0.822** | 0.915 | 0.791 | 0.848 |
| PacBio  CLR 10x | SVDF | 0.887 | 0.703 | 0.784 | 0.836 | 0.573 | 0.680 | 0.859 | 0.626 | **0.724** |
|  | SVIM | 0.883 | 0.671 | 0.763 | 0.750 | 0.458 | 0.569 | 0.812 | 0.546 | 0.653 |
|  | Sniffles2 | 0.855 | 0.738 | **0.792** | 0.435 | 0.634 | 0.516 | 0.557 | 0.676 | 0.611 |
|  | cuteSV2 | 0.889 | 0.674 | 0.767 | 0.829 | 0.579 | **0.682** | 0.855 | 0.618 | 0.717 |
| PacBio  CLR 5x | SVDF | 0.819 | 0.551 | **0.659** | 0.563 | 0.406 | 0.472 | 0.664 | 0.466 | 0.547 |
|  | SVIM | 0.843 | 0.323 | 0.467 | 0.731 | 0.196 | 0.309 | 0.787 | 0.248 | 0.377 |
|  | Sniffles2 | 0.787 | 0.502 | 0.613 | 0.382 | 0.440 | 0.409 | 0.495 | 0.466 | 0.480 |
|  | cuteSV2 | 0.804 | 0.517 | 0.629 | 0.572 | 0.447 | **0.502** | 0.656 | 0.476 | **0.552** |
| PacBio  CCS 30x | SVDF | 0.952 | 0.950 | **0.951** | 0.908 | 0.910 | **0.909** | 0.926 | 0.926 | **0.926** |
|  | SVIM | 0.923 | 0.940 | 0.931 | 0.827 | 0.871 | 0.848 | 0.865 | 0.899 | 0.882 |
|  | Sniffles2 | 0.922 | 0.937 | 0.930 | 0.869 | 0.906 | 0.887 | 0.891 | 0.919 | 0.905 |
|  | cuteSV2 | 0.948 | 0.925 | 0.936 | 0.917 | 0.900 | 0.908 | 0.929 | 0.910 | 0.919 |
| PacBio  CCS 10x | SVDF | 0.923 | 0.875 | **0.899** | 0.865 | 0.811 | **0.837** | 0.889 | 0.837 | **0.862** |
|  | SVIM | 0.902 | 0.776 | 0.834 | 0.833 | 0.725 | 0.775 | 0.861 | 0.746 | 0.799 |
|  | Sniffles2 | 0.880 | 0.829 | 0.854 | 0.846 | 0.819 | 0.832 | 0.860 | 0.823 | 0.841 |
|  | cuteSV2 | 0.882 | 0.799 | 0.838 | 0.874 | 0.798 | 0.834 | 0.877 | 0.798 | 0.836 |
| PacBio  CCS 5x | SVDF | 0.858 | 0.766 | **0.809** | 0.804 | 0.728 | 0.764 | 0.826 | 0.744 | **0.783** |
|  | SVIM | 0.843 | 0.378 | 0.522 | 0.812 | 0.357 | 0.496 | 0.825 | 0.366 | 0.507 |
|  | Sniffles2 | 0.835 | 0.613 | 0.707 | 0.880 | 0.829 | **0.854** | 0.832 | 0.630 | 0.717 |
|  | cuteSV2 | 0.789 | 0.640 | 0.707 | 0.828 | 0.707 | 0.763 | 0.813 | 0.680 | 0.740 |
| Oxford  Nanopore 50x | SVDF | 0.941 | 0.949 | **0.945** | 0.897 | 0.840 | **0.868** | 0.916 | 0.884 | **0.900** |
|  | SVIM | 0.902 | 0.919 | 0.911 | 0.624 | 0.625 | 0.624 | 0.738 | 0.746 | 0.742 |
|  | Sniffles2 | 0.893 | 0.934 | 0.913 | 0.821 | 0.818 | 0.820 | 0.852 | 0.865 | 0.858 |
|  | cuteSV2 | 0.933 | 0.925 | 0.929 | 0.876 | 0.831 | 0.853 | 0.900 | 0.870 | 0.885 |
| Oxford  Nanopore 20x | SVDF | 0.925 | 0.918 | **0.921** | 0.864 | 0.776 | 0.817 | 0.890 | 0.834 | **0.861** |
|  | SVIM | 0.871 | 0.913 | 0.892 | 0.551 | 0.634 | 0.589 | 0.675 | 0.748 | 0.710 |
|  | Sniffles2 | 0.858 | 0.915 | 0.886 | 0.795 | 0.796 | 0.795 | 0.822 | 0.845 | 0.833 |
|  | cuteSV2 | 0.916 | 0.893 | 0.905 | 0.849 | 0.803 | **0.825** | 0.877 | 0.840 | 0.858 |
| Oxford  Nanopore 10x | SVDF | 0.872 | 0.814 | **0.842** | 0.810 | 0.671 | 0.734 | 0.837 | 0.730 | **0.780** |
|  | SVIM | 0.840 | 0.804 | 0.821 | 0.562 | 0.550 | 0.556 | 0.674 | 0.654 | 0.664 |
|  | Sniffles2 | 0.810 | 0.842 | 0.826 | 0.749 | 0.720 | 0.734 | 0.775 | 0.770 | 0.773 |
|  | cuteSV2 | 0.865 | 0.782 | 0.821 | 0.798 | 0.686 | **0.737** | 0.826 | 0.725 | 0.772 |
| Oxford  Nanopore 5x | SVDF | 0.768 | 0.643 | **0.700** | 0.736 | 0.546 | **0.627** | 0.750 | 0.586 | **0.658** |
|  | SVIM | 0.838 | 0.433 | 0.571 | 0.602 | 0.306 | 0.406 | 0.700 | 0.358 | 0.474 |
|  | Sniffles2 | 0.762 | 0.636 | 0.693 | 0.698 | 0.551 | 0.616 | 0.725 | 0.586 | 0.648 |
|  | cuteSV2 | 0.757 | 0.606 | 0.673 | 0.710 | 0.541 | 0.614 | 0.730 | 0.567 | 0.638 |

Table S6 Benchmark results on HG002 sample from CMRG ground-truth

| Dataset | Tool | P | R | F1 | P-GT | R-GT | F1-GT |
| --- | --- | --- | --- | --- | --- | --- | --- |
| PacBio  CLR 65x | SVDF | 0.975 | 0.796 | **0.876** | 0.813 | 0.663 | **0.730** |
|  | SVIM | 0.986 | 0.735 | 0.842 | 0.849 | 0.633 | 0.725 |
|  | Sniffles2 | 0.889 | 0.816 | 0.851 | 0.733 | 0.673 | 0.702 |
|  | cuteSV2 | 0.951 | 0.786 | 0.860 | 0.765 | 0.633 | 0.693 |
| PacBio  CCS 30x | SVDF | 0.966 | 0.867 | 0.914 | 0.852 | 0.76 | 0.806 |
|  | SVIM | 0.955 | 0.867 | 0.909 | 0.843 | 0.765 | 0.802 |
|  | Sniffles2 | 0.966 | 0.878 | **0.920** | 0.865 | 0.786 | **0.824** |
|  | cuteSV2 | 0.976 | 0.837 | 0.901 | 0.833 | 0.714 | 0.769 |
| Oxford  Nanopore 50x | SVDF | 0.928 | 0.786 | **0.851** | 0.819 | 0.694 | **0.751** |
|  | SVIM | 0.755 | 0.755 | 0.755 | 0.663 | 0.663 | 0.663 |
|  | Sniffles2 | 0.893 | 0.765 | 0.824 | 0.726 | 0.622 | 0.670 |
|  | cuteSV2 | 0.880 | 0.745 | 0.807 | 0.771 | 0.65 | 0.700 |

Table S7 Benchmark results on Ashkenazi human trio

| SV type | SVDF | | | SVIM | | | Sniffles2 | | | cuteSV2 | | |
| --- | --- | --- | --- | --- | --- | --- | --- | --- | --- | --- | --- | --- |
|  | Total  calls | Not in parents | MDR  (%) | Total calls | Not in parents | MDR  (%) | Total calls | Not in parents | MDR  (%) | Total calls | Not in parents | MDR  (%) |
| DEL | 3406 | 128 | **3.76** | 4553 | 321 | 7.05 | 2992 | 124 | 4.14 | 4119 | 284 | 6.89 |
| INS | 4847 | 466 | 9.61 | 5934 | 514 | **8.66** | 4898 | 564 | 11.51 | 5924 | 637 | 10.75 |
| DUP | 298 | 34 | **11.41** | 35 | 4 | 11.43 | 156 | 28 | 17.95 | 381 | 78 | 20.47 |
| INV | 16 | 0 | **0** | 47 | 4 | 8.51 | 93 | 22 | 23.66 | 50 | 15 | 30.00 |
| TRA | 429 | 176 | 41.03 | - | - | - | 422 | 183 | 43.36 | 440 | 37 | **8.41** |
| Total | 8996 | 804 | 8.94 | 10571 | 843 | 7.97 | 8561 | 921 | 10.76 | 10914 | 1051 | 9.63 |

Table S8 Benchmark results of SV call and genotype on CHM13 sample

| SV type | Tool | Total calls | TP-call | P | R | F1 | P-GT | R-GT | F1-GT |
| --- | --- | --- | --- | --- | --- | --- | --- | --- | --- |
| DEL | SVDF | 6206 | 4954 | 0.798 | 0.74 | 0.77 | 0.690 | 0.644 | 0.666 |
|  | SVIM | 6644 | 5132 | 0.77 | 0.771 | **0.772** | 0.47 | 0.471 | 0.47 |
|  | Sniffles2 | 7066 | 5097 | 0.721 | 0.766 | 0.743 | 0.663 | 0.704 | **0.683** |
|  | cuteSV2 | 5648 | 4176 | 0.739 | 0.62 | 0.679 | 0.691 | 0.587 | 0.635 |
| INS | SVDF | 9477 | 7610 | 0.803 | 0.713 | **0.755** | 0.707 | 0.627 | **0.665** |
|  | SVIM | 9278 | 6985 | 0.753 | 0.654 | 0.700 | 0.33 | 0.293 | 0.313 |
|  | Sniffles2 | 10167 | 7453 | 0.733 | 0.69 | 0.71 | 0.65 | 0.62 | 0.638 |
|  | cuteSV2 | 7847 | 5726 | 0.730 | 0.536 | 0.618 | 0.712 | 0.523 | 0.603 |
| Total | SVDF | 15683 | 12564 | 0.801 | 0.725 | **0.761** | 0.7 | 0.634 | **0.665** |
|  | SVIM | 15922 | 12117 | 0.761 | 0.699 | 0.729 | 0.39 | 0.361 | 0.377 |
|  | Sniffles2 | 17233 | 12550 | 0.728 | 0.724 | 0.726 | 0.658 | 0.654 | 0.656 |
|  | cuteSV2 | 13495 | 9902 | 0.734 | 0.57 | 0.642 | 0.703 | 0.547 | 0.616 |

Table S9 Benchmark results of SV breakpoint deviation (0, 10, 20 bp) on CHM13 sample

| SV type | tools | 0 | | | | 10 | | | | 20 | | | |
| --- | --- | --- | --- | --- | --- | --- | --- | --- | --- | --- | --- | --- | --- |
|  |  | TP | P | R | F1 | TP | P | R | F1 | TP | P | R | F1 |
| DEL | SVDF | 4815 | 0.776 | 0.724 | **0.749** | 4837 | 0.779 | 0.727 | **0.75**2 | 4852 | 0.782 | 0.729 | 0.755 |
|  | SVIM | 5015 | 0.755 | 0.754 | 0.754 | 5043 | 0.759 | 0.758 | 0.758 | 5065 | 0.762 | 0.761 | **0.762** |
|  | Sniffles2 | 4966 | 0.703 | 0.746 | 0.724 | 4989 | 0.706 | 0.750 | 0.727 | 5005 | 0.708 | 0.752 | 0.730 |
|  | cuteSV2 | 4044 | 0.716 | 0.608 | 0.657 | 4066 | 0.720 | 0.611 | 0.661 | 4084 | 0.723 | 0.614 | 0.664 |
| INS | SVDF | 4642 | 0.490 | 0.435 | **0.461** | 5869 | 0.619 | 0.550 | **0.583** | 6354 | 0.670 | 0.595 | **0.631** |
|  | SVIM | 595 | 0.064 | 0.056 | 0.060 | 4196 | 0.452 | 0.393 | 0.421 | 5099 | 0.550 | 0.478 | 0.511 |
|  | Sniffles2 | 32 | 0.003 | 0.003 | 0.003 | 5889 | 0.579 | 0.552 | 0.565 | 6233 | 0.613 | 0.584 | 0.598 |
|  | cuteSV2 | 1608 | 0.205 | 0.151 | 0.174 | 2943 | 0.375 | 0.276 | 0.318 | 2943 | 0.451 | 0.332 | 0.383 |
| Total | SVDF | 9457 | 0.603 | 0.546 | **0.573** | 10706 | 0.683 | 0.618 | **0.649** | 11206 | 0.715 | 0.647 | **0.679** |
|  | SVIM | 5610 | 0.352 | 0.324 | 0.337 | 9239 | 0.580 | 0.533 | 0.556 | 10164 | 0.638 | 0.587 | 0.611 |
|  | Sniffles2 | 4998 | 0.290 | 0.288 | 0.289 | 10878 | 0.631 | 0.628 | 0.629 | 11238 | 0.652 | 0.649 | 0.650 |
|  | cuteSV2 | 5652 | 0.419 | 0.326 | 0.367 | 7008 | 0.519 | 0.404 | 0.455 | 7625 | 0.565 | 0.440 | 0.495 |

Table S10 Call number of cancer-specific SV in HCC_1395_ sample

| Dataset | SV type | SVDF | SVIM | Sniffles2 | cuteSV2 | SVDF (verified) | SVIM (verified) | Sniffles2  (verified) | cuteSV2 (verified) |
| --- | --- | --- | --- | --- | --- | --- | --- | --- | --- |
| PacBio CLR | DEL | 591 | 573 | 572 | 581 | 71 | 48 | 52 | 55 |
|  | INS/DUP | 692 | 625 | 639 | 623 | 68 | 28 | 41 | 28 |
|  | INV | 50 | 13 | 43 | 28 | 17 | 2 | 16 | 3 |
|  | TRA | 68 | 76 | 51 | 61 | 36 | 37 | 24 | 34 |
| Oxford  Nanopore | DEL | 265 | 280 | 251 | 278 | 0 | 0 | 0 | 0 |
|  | INS/DUP | 448 | 436 | 445 | 456 | 1 | 1 | 1 | 1 |
|  | INV | 6 | 3 | 2 | 4 | 0 | 0 | 0 | 0 |
|  | TRA | 1 | 0 | 0 | 1 | 0 | 0 | 0 | 0 |
| CLR+ONT  (1788) | DEL | **608** | 589 | 596 | 601 | **71** | 48 | 52 | 55 |
|  | INS/DUP | **726** | 662 | 672 | 660 | **68** | 28 | 41 | 28 |
|  | INV | **53** | 13 | 43 | 29 | **17** | 2 | 16 | 3 |
|  | TRA | 68 | **76** | 51 | 61 | 36 | **37** | 24 | 34 |
|  | Total | **1455** | 1340 | 1362 | 1351 | **192** | 115 | 133 | 120 |

Table S11 Call result of cancer-specific SV in HCC_1395_ sample from Severus benchmark set

| Dataset | Tools | Total calls | TP | Recall (Minda) | Recall (Truvari) |
| --- | --- | --- | --- | --- | --- |
| PacBio CLR  (1011) | SVDF | 50517 | 936 | **0.926** | **0.871** |
|  | SVIM | 30034 | 822 | 0.813 | 0.783 |
|  | Sniffles2 | 30576 | 691 | 0.683 | 0.684 |
|  | cuteSV2 | 37641 | 693 | 0.685 | 0.683 |
| Oxford Nanopore  (3467) | SVDF | 67248 | 2907 | **0.838** | **0.822** |
|  | SVIM | 80970 | 2747 | 0.792 | 0.727 |
|  | Sniffles2 | 22439 | 2564 | 0.740 | 0.681 |
|  | cuteSV2 | 20747 | 2310 | 0.666 | 0.611 |

Table S12 Runtime and memory usage in HG002 HIFI dataset (30X)

| Tools | Threads | Elapsed runtime (min) | Max memory consumption (MB) |
| --- | --- | --- | --- |
| SVDF | 16 | 5.15 | 8,764,064 |
| SVIM | 1 | 16.32 | 988,920 |
| Sniffles2 | 16 | **2.03** | **484,036** |
| cuteSV2 | 16 | 2.28 | 1,780,448^^[[2]](#footnote-2)^^ |
| MAMnet | 16 | 18.20 | 55,538,864 |

Runtime and memory footprint were assessed by using the "/usr/bin/time -v" command of the Linux Operating System. SVIM does not support multithreading. MAMnet (<https://github.com/micahvista/MAMnet>) is a representative SV calling method using deep learning.

Table S13 Data availability

| Data | Link |
| --- | --- |
| The sequencing reads and bam data of HG002 | <https://github.com/genome-in-a-bottle/giab_data_indexes> |
| The tier1 benchmark SV call set and high-confidence region of HG002 | [https://ftp.ncbi.nih.gov/giab/ftp/data/AshkenazimTrio/analysis/NIST_SVs_Integration_v0.6](https://ftp.ncbi.nih.gov/giab/ftp/data/AshkenazimTrio/analysis/NIST_SVs_Integration_v0.6/) |
| The Ashkenazim trio (including HG002, HG003, and HG004) sequencing reads and bam data | <https://ftp.ncbi.nih.gov/giab/ftp/data/AshkenazimTrio/> |
| The Nanopore reads and bam data of CHM13 | <https://github.com/marbl/CHM13> |
| The assembly-based SV call sets of CHM13 | <https://github.com/ldenti/SVDSS-experiments> |
| The PacBio CLR and Nanopore reads of the HCC_1395_ and HCC_1395_BL | <http://ftp-trace.ncbi.nlm.nih.gov/ReferenceSamples/seqc/Somatic_Mutation_WG> |
| The cancer-specific benchmark SV call sets | <https://doi.org/10.1186/s13059-022-02816-6> |

**Supplementary Notes**

1. Implementation of training dataset and simulated sample generation

The training data for the intra-alignment signatures were derived from the HG002 sample, which covered different sequencing platforms and alignment tools, aiming to improve the generalisation performance of the model. We collected intra- and inter-alignment signatures from all BAM files, filtered out signatures outside the high-confidence regions released by GIAB, and used the ground truth (HG002_SVs_Tier1_v0.6.vcf) for signature annotation. Two signatures originating from DEL/INS/DUP/INV types on the same chromosome were considered part of the same SV event if they satisfied the following equation:

$\left\{\begin{matrix} \left|s_{1}-s_{2}\right|\leq1000 \\ \frac{\min(size_{1},size_{2})}{\max(size_{1},size_{2})}\geq0.5 \end{matrix}\right.$ (1)

where *s_1_, s_2_, size_1_*, and *size_2_* represent the starting coordinates and lengths of the signatures, respectively. For translocation, if the breakpoints of two signatures share the same chromosome and are within 1000 bp, they are considered to be the same SV event. Following the above criteria, we performed a binary search on the ground truth set. If a corresponding SV event was found in the ground truth set, the signature was labelled with the corresponding SV category, and signatures not found in the ground truth set were labelled false case. For the intra-alignment signature clusters, the label of each cluster (DEL/INS/false case) was determined from the voting results of the labels of each signature within the cluster.

Due to the HG002 sample benchmark set released by GIAB included only two types of SVs (deletions and insertions), we subsequently used the SURVIVOR tool to simulate 14,000 SV events covering five SV types, DEL/INS/DUP/INV/TRA, with corresponding proportions of 5000:5000:2000:1000:1000. These SV events were randomly inserted into the hs37d5 reference genome, and 30X PacBio and Nanopore reads were generated using the PBSIM2 tool. Alignment files were generated using the minimap2 tool based on the hs37d5 reference genome. The inter-alignment signatures in the BAM files generated from the simulated data were annotated based on the SV benchmark set in the bed format produced by SUVIVOR and Equation (1). Subsequently, these annotations were merged with inter-alignment signatures from the DEL/INS events of the HG002 sample to create a comprehensive inter-alignment signature set.

Finally, the signature data from chromosomes to 1-11 in the two generated signature sets were randomly divided into training and validation sets in a 7:3 ratio, and the test set comprised signature data from chromosomes to 12-22, X, and Y.

1. Implementation of SV calling

In this study, the hs37d5 version was used as the reference genome for both the HG002 and simulated samples, whereas the GRCh38 version was used for the CHM13 and HCC_1395_ samples. Multiple alignment tools were employed to generate the BAM files. For the CLR, CCS, and ONT sequencing data of the HG002 sample, three alignment tools, NGMLR, PBMM2, and minimap2, were individually used for alignment. Alignment data for the remaining samples were aligned using the minimap2 tool.

Three state-of-the-art SV detection tools, SVIM (v1.4.2), Sniffles2 (v2.2), and cuteSV2 (v2.1.0), were employed for experimental comparisons using the default or recommended settings. Apart from Sniffles2, all other tools employ a manual setting of a minimum supporting read threshold to filter low-quality SVs. The minimum supporting read parameter was uniformly set based on sequencing depth and data type. Detailed command lines for the implementation can be found in Section 4.

1. Evaluation of SV calling

The SURVIVOR tool (v1.0.3) was used to evaluate the results generated for the simulated samples using the eval command (with the maximum offset error set to 500 bp). The precision, recall, and F1 score were then calculated based on the true positive (TP), false negative (FN), and false positive (FP) values reported by SURVIVOR.

The Truvari tool (v3.1) was used to evaluate the results generated for both HG002 and CHM13 real samples. The SV benchmark set and high-confidence regions of HG002 was derived from GIAB, whereas the SV benchmark set of CHM13 was obtained from the SV calling set generated from de novo assembly using Dipcall, as provided in the recent work SVDSS.

We used the SV call set from the Ashkenazi Trio to evaluate the MDR. The sample data for parents HG003 and HG004 were obtained from the alignment files of 30X PacBio CLR sequencing released by GIAB. For the offspring sample HG002, we selected the alignment file from 35X PacBio CLR sequencing, which has a sequencing coverage similar to that of the parental samples. Therefore, MDR was calculated as the percentage of SVs in the HG002 sample that could not be detected in the HG003 and HG004 samples, where *SV_discordant_* was calculated according to equation (1):

$MDR=\frac{\sum SV_{discordant}}{\sum SV_{offspring}}$ (2)

To evaluate the HCC1395 sample results, Equation (1) was used to calculate the number of SVs recalled in the cancer-specific SV benchmark set released by the SEQC2 consortium for all SV call sets. The Minda tool was used to evaluate the recall based on the benchmark set by Severus.

1. Commands used for benchmark

**4.1 SV simulate**

./SURVIVOR simSV parameter_file

PARAMETER FILE:

PARAMETER FILE: DO JUST MODIFY THE VALUES AND KEEP THE SPACES!

DUPLICATION_minimum_length: 100

DUPLICATION_maximum_length: 5000

DUPLICATION_maximum_num: 5

DUPLICATION_number: 2000

INDEL_minimum_length: 40

INDEL_maximum_length: 1000

INDEL_number: 10000

TRANSLOCATION_minimum_length: 1000

TRANSLOCATION_maximum_length: 5000

TRANSLOCATION_number: 1000

INVERSION_minimum_length: 600

INVERSION_maximum_length: 800

INVERSION_number: 1000

INV_del_minimum_length: 600

INV_del_maximum_length: 800

INV_del_number: 0

INV_dup_minimum_length: 600

INV_dup_maximum_length: 800

INV_dup_number: 0

Number_haploid: 1

homozygous_ratio: 0.6

./SURVIVOR simSV ref.fa parameter_file 0.1 0 simulated

pbsim --depth 30 --hmm_model R103.model simulated.fasta

pbsim --depth 30 --hmm_model P6C4.model simulated.fasta

**4.2 Read alignment**

minimap2 -t 64 -ax map-ont hs37d5.fa output.fastq > aln.sam

samtools view -@ 64 -u aln.sam | samtools sort -@ 64 -T aln.bam.sort-tmp > aln.bam

samtools index aln.bam

- 1. **SV call**

SVDF:

python svdf.py call data/test.bam --working_dir ./ -s {min-support} -t 16;

sensitive mode: python svdf.py call data/test.bam --mode sensitive --working_dir ./ -s {min-support}

SVIM:

svim alignment tools/svim/variants.vcf data/test.bam ref/hs37d5.fa --min_sv_size 30;

cat tools/svim/variants.vcf | sed 's/DUP:TANDEM/DUP/g' > tools/svim/variants.vcf

Sniffles2:

sniffles --input data/test.bam --vcf tools/sniffles/variants.vcf --allow-overwrite -t 16

cuteSV2:

cuteSV data/test.bam ref/hs37d5.fa tools/cutesv/variants.vcf ./ -s {min-support}--genotype -t 16;

ONT: --max_cluster_bias_INS 100 --diff_ratio_merging_INS 0.3 --max_cluster_bias_DEL 100 --diff_ratio_merging_DEL 0.3;

CLR: --max_cluster_bias_INS 100 --diff_ratio_merging_INS 0.3 --max_cluster_bias_DEL 200 --diff_ratio_merging_DEL 0.5;

CCS: --max_cluster_bias_INS 1000 --diff_ratio_merging_INS 0.9 --max_cluster_bias_DEL 1000 --diff_ratio_merging_DEL 0.5;

Severus:

python severus.py --target-bam data/test_tumor.bam --control-bam data/normal.bam --out-dir tools/severus/ --vntr-bed Severus/vntrs/human_GRCh38_no_alt_analysis_set.trf.bed --min-support 5 -t 16

The "min-support" parameter of supporting reads note:

For the HG002 sample's CLR 69X/35X/20X/10X/5X data, supporting reads were set to 10/5/4/3/2, respectively. For the HG002 sample's CCS 28X/10X/5X data, supporting reads were set to 3/2/1. For the HG002 sample's ONT 50X/20X/10X/5X data, supporting reads were set to 10/4/3/2. For the CHM13 sample's ONT 126X data, supporting reads were set to 10. For simulated data CLR 30X and ONT 30X, supporting reads were set to 5 for both. For the HCC_1395_ sample's CLR 39X data and ONT 12X data, supporting reads were set to 5 and 3, respectively.

**4.4 SV eval**

Truvari:

bgzip {variants.vcf} && tabix {variants.vcf.gz};

truvari bench -f ref/hs37d5.fa -b giab/HG002_SVs_Tier1_v0.6.vcf.gz -o {tool_eval} --sizemin 50 --sizefilt 50 --passonly -p 0.00 -c variants.vcf.gz --includebed giab/HG002_SVs_Tier1_v0.6.bed

SURVIVOR:

SURVIVOR eval variants.vcf sim.bed 500 eval_res

Minda:

python minda.py truthset --base --min_size 50 --vcfs caller_1.vcf caller_2.vcf caller_3.vcf caller_4.vcf --out_dir minda_out

1. [↑](#footnote-ref-1)
2. [↑](#footnote-ref-2)
